# On Kiefer’s American *Eucyclops* (Copepoda, Eucyclopinae): redescriptions and comments on the historical records of *E. delachauxi*, *E. prionophorus*, *E. bondi* and *E. leptacanthus*

**DOI:** 10.3897/zookeys.402.6112

**Published:** 2014-04-16

**Authors:** Nancy F. Mercado-Salas, Eduardo Suárez-Morales

**Affiliations:** 1 El Colegio de la Frontera Sur (ECOSUR). Unidad Chetumal. Av. Centenario Km. 5.5. Chetumal, Quintana Roo 77014. México

**Keywords:** Freshwater copepods, Eucyclopinae, morphology, systematics, taxonomy

## Abstract

The freshwater copepod genus *Eucyclops* contains many supposedly cosmopolitan species whose taxonomic status is still under discussion; some of them represent species complexes. The problem is not exclusive to these widespread species; there are several American *Eucyclops* needing a taxonomic re-evaluation. Based on the examination of Friedrich Kiefer’s collection in Karlsruhe, Germany, the type specimens of four American species of *Eucyclops* (*E. delachauxi* (Kiefer, 1926), *E. prionophorus* Kiefer, 1931, *E. bondi* Kiefer, 1934, *E. leptacanthus* Kiefer, 1956) were re-examined and redescribed using upgraded descriptive standards. Kiefer’s translated descriptions and unpublished original drawings of these species are also presented. Characters like the ornamentation of the antennal basis, ornamentation of intercoxal sclerites of the swimming legs 1–4, length of basipodal seta of leg 1, ornamentation of caudal rami, the presence of aesthetascs and modified setae on the antennules in male, and the structure of the male sixth leg are compared herein to aid a more accurate separation of these American species. A revision of the American records of these species confirms that some are likely to refer to undescribed species. Overall, the diversity of the American *Eucyclops* appears to be underestimated and certainly deserves further study.

## Introduction

The freshwater genus *Eucyclops* Claus, 1893 is currently known to contain more than 108 species and subspecies (Alekseev and Defaye 2011), thus being one of the most speciose genera among the Cyclopoida. Only a few species have been completely described following upgraded standards; in addition, the genus taxonomy was based, until recent years, on a small number of highly variable characters. Consequently, *Eucyclops* has a complex taxonomic history that includes several widely distributed species with an uncertain status (Collado et al. 1984; Reid 1985; Ishida 1997; Suárez-Morales 2004; Mercado-Salas et al. 2012).

The taxonomic problems within this taxon started with the incomplete description of the type species of the genus: *Eucyclops serrulatus* (Fisher, 1851) from Russia (Alekseev et al. 2006). One of the first researchers in pointing out these deficiencies was Friedrich Kiefer. In his description of *Eucyclops delachauxi* (Kiefer, 1925) and in a subsequent paper on the Peruvian copepod fauna (Kiefer 1926) he stated that the systematics and geographical distribution of this group were far from being understandable if every single *Cyclops* with “*serra*” was identified as *Eucyclops serrulatus* and if other characters (besides the “*serra*”) were not incorporated into the delimitation of species. He also noticed that the *serrulatus*-group as a whole, rather than its member taxa, was cosmopolitan and ubiquitous as it had been previously assumed. After Kiefer’s studies, many species were described all over the world, but most scientists further continued using a reduced number of variable characters only. The morphological definition of *Eucyclops serrulatus* and its cosmopolitan status remained unchallenged until recent years. Alekseev (1990, 2008, 2010), Ishida (1997, 2001, 2002, 2003), Alekseev et al. (2006), and Alekseev and Defaye (2011) have been the pioneers solving the taxonomical problems among the *Eucyclops* taxa, with the delimitation of the “*serrulatus*-like” and “*speratus*-like” species from Japan, and the *serrulatus*-group worldwide. The comparison of new characters such as the ornamentation of the antennal basis, the ornamentation of swimming legs (especially the fourth), and the integumental pore signature have revealed consistent differences among species, which were previously overlooked and should be verified in the rest of the species of the genus.

In a project to explore the species diversity of the genus *Eucyclops* in Mexico, the type material of some of these species was examined. There are four species described by Kiefer, which have been recorded in Mexico: *Eucyclops delachauxi* (Kiefer, 1925), *Eucyclops prionophorus* Kiefer, 1931, *Eucyclops bondi* Kiefer, 1934 and *Eucyclops leptacanthus* Kiefer, 1956 (Suárez-Morales and Reid 1998, Grimaldo-Ortega et al. 1998; Elías-Gutiérrez 2000; Rodríguez-Almaraz 2000; Suárez-Morales 2004, Mercado-Salas 2009; Suárez-Morales et al. 2010; Mercado-Salas and Suárez-Morales 2012). In order to clarify the taxonomic identity of the Mexican material, the type specimens were examined at the Staatliches Museum für Naturkunde, Karlsruhe (Germany) where F. Kiefer’s collection is held. Herein we present the redescription of the four species mentioned above using upgraded standards; we also include Kiefer’s unpublished original illustrations. In addition, we provide English translations of the original descriptions, in order to make Kiefer’s detailed observations and complementary unpublished data available.

## Methods

In order to provide an upgraded morphological redescription of *Eucyclops delachauxi*, *Eucyclops prionophorus*, *Eucyclops bondi*, and *Eucyclops leptacanthus*, we examined the type material of Kiefer’s collection deposited at the Staatliches Museum für Naturkunde Karlsruhe (Germany). Drawings were made at 1000× with a Zeiss Axioskop 2 plus compound microscope equipped with a camera lucida. Mapping of rows of spinules and setules on the antennal basis and on the coxopodite and intercoxal slerite of P4 followed Alekseev et al. (2006) and Alekseev and Defaye (2011). Abbreviations used in the descriptive section are as follows: P1-P4, first to fourth thoracic limbs; Exp, exopod; Enp, endopod; s, seta(e); ae, aesthetasc; sp, spine; Bsp, basis; Fu, caudal ramus. Nomenclature used for armament of the antennule and antenna followed Alekseev et al. (2006) and Alekseev and Defaye (2011). Caudal seta nomenclature as follows: II – anterolateral (lateral) caudal seta; III – posterolateral (outermost) caudal seta; IV – outer terminal (terminal median external) caudal seta; V – inner terminal (terminal median internal) caudal seta; VI – terminal accessory (innermost) caudal seta; VII – dorsal seta.

## Results

For each of these four species we present first the complete translation from German to English of Kiefer’s description, followed by an upgraded description based on our personal observations on Kiefer’s material. Characters or structures not observed but previously published by other researchers are included in the descriptions with its reference. Figures mentioned in the translated text correspond to the numbers of the figures in the original descriptions published by Kiefer (1925, 1931, 1934, 1956).

### Order Cyclopoida Rafinesque, 1815
Family Cyclopidae Rafinesque, 1815
Subfamily Eucyclopinae Kiefer, 1927
Genus *Eucyclops* Claus, 1893
*Eucyclops delachauxi* (Kiefer, 1925)

Figs 1–7

*Cyclops delachauxi* (Kiefer, 1925)

*Cyclops delachauxi* (Kiefer, 1926)

*Eucyclops (Eucyclops) delachauxi* Kiefer, 1929

*Cyclops Delachauxi* Kiefer, 1925

*Cyclops Delachauxi* Kiefer, 1926

*Eucyclops (Eucyclops) Delachauxi*, Kiefer, 1929

*Eucyclops Delachauxi* Kiefer, 1943

*Eucyclops delachauxi* Lindberg, 1955, 1957

**Kiefer’s description.**

**Figure 1.**
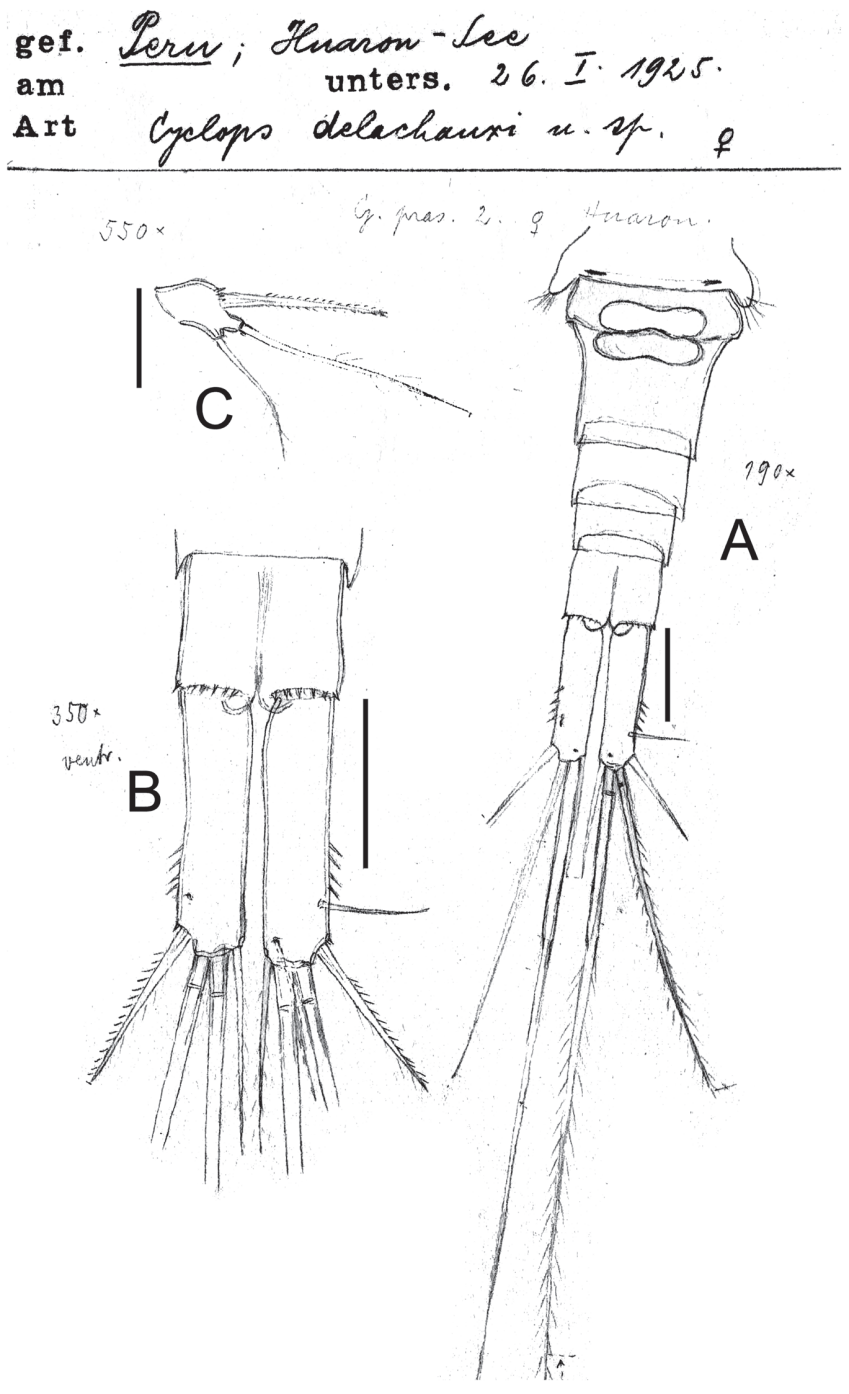
*Eucyclops delachauxi* (Kiefer, 1925). Original drawings of F. Kiefer. Female Holotype from Huaron, Peru. **A** Urosome, ventral view **B** Caudal rami **C** P5. Scale bars: **A–B** = 50 µm, **C** = 20 µm.

**Figure 2.**
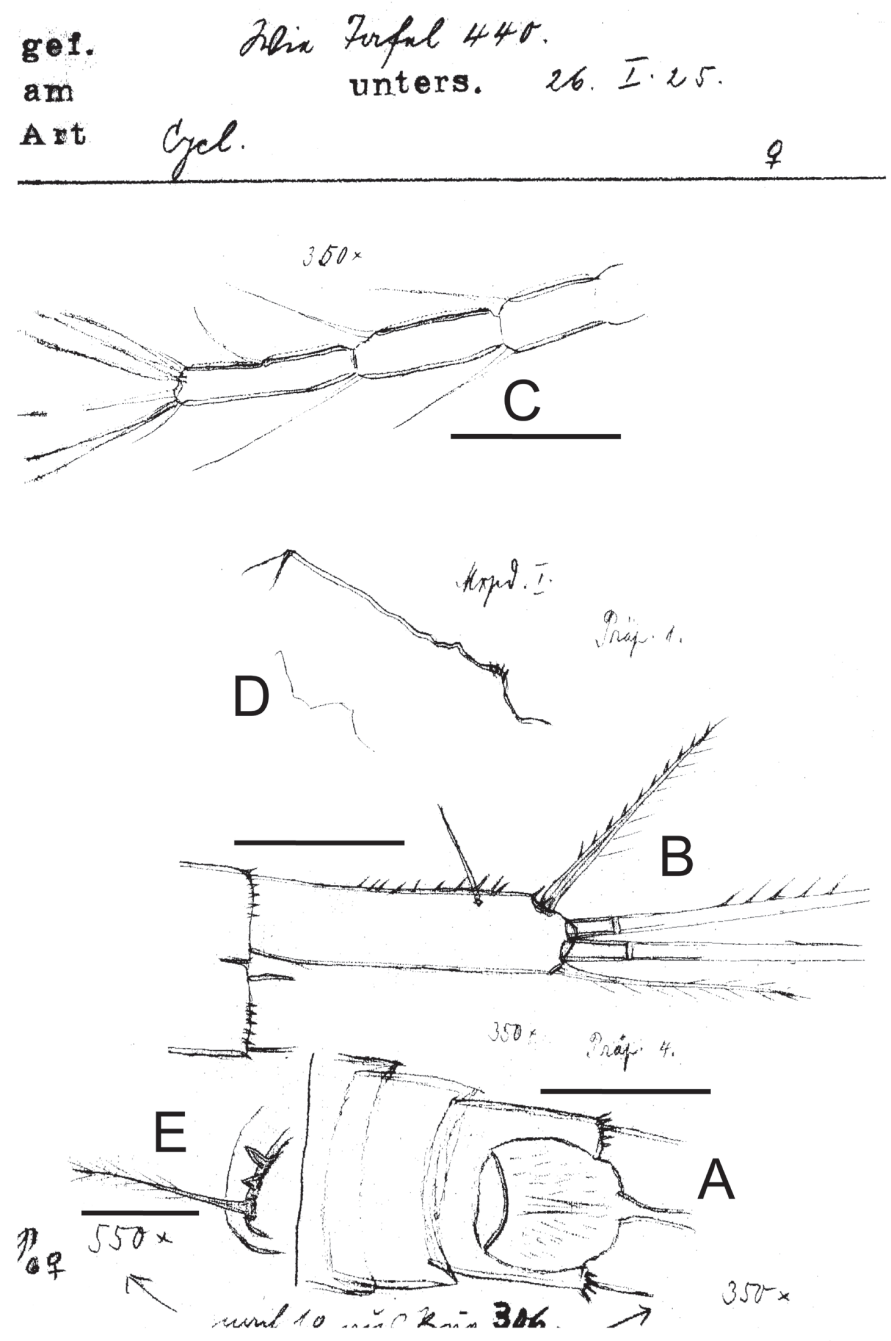
*Eucyclops delachauxi* (Kiefer, 1925). Original drawings of F. Kiefer. Female Holotype from Huaron, Peru. **A** Anal somite, dorsal view **B** Caudal rami **C** Antennule, segments 10–12 **D** Praecoxa of maxilla **E** P6. Scale bars: **A–D** = 50 µm, **E** = 20 µm.

**Figure 3.**
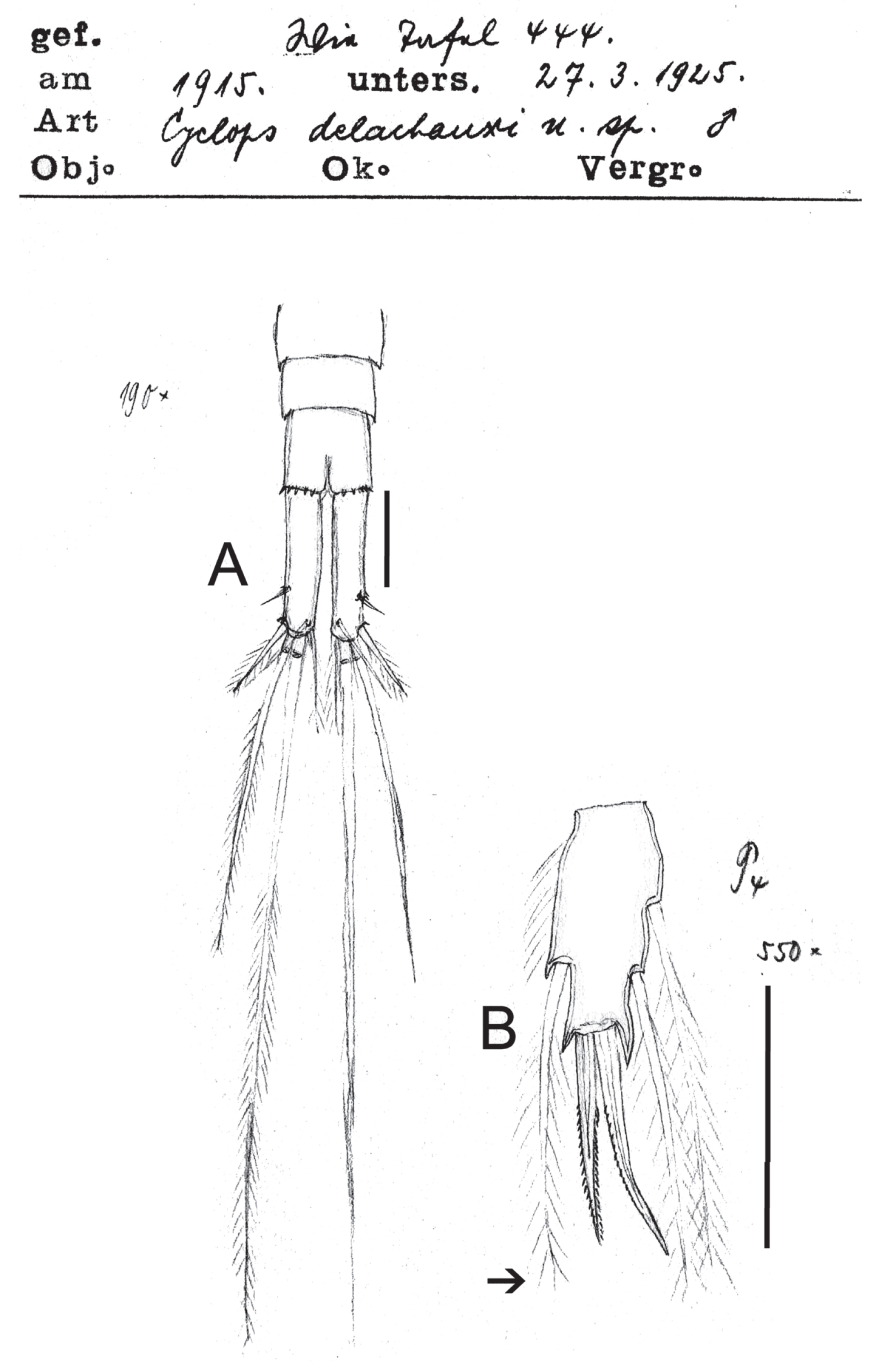
*Eucyclops delachauxi* (Kiefer, 1925). Original drawings of F. Kiefer. Paratype from Huaron, Peru. **A** Last urosomites and furca **B** Enp3P4. Scale bars: **A–B** = 50 µm.

**Figure 4.**
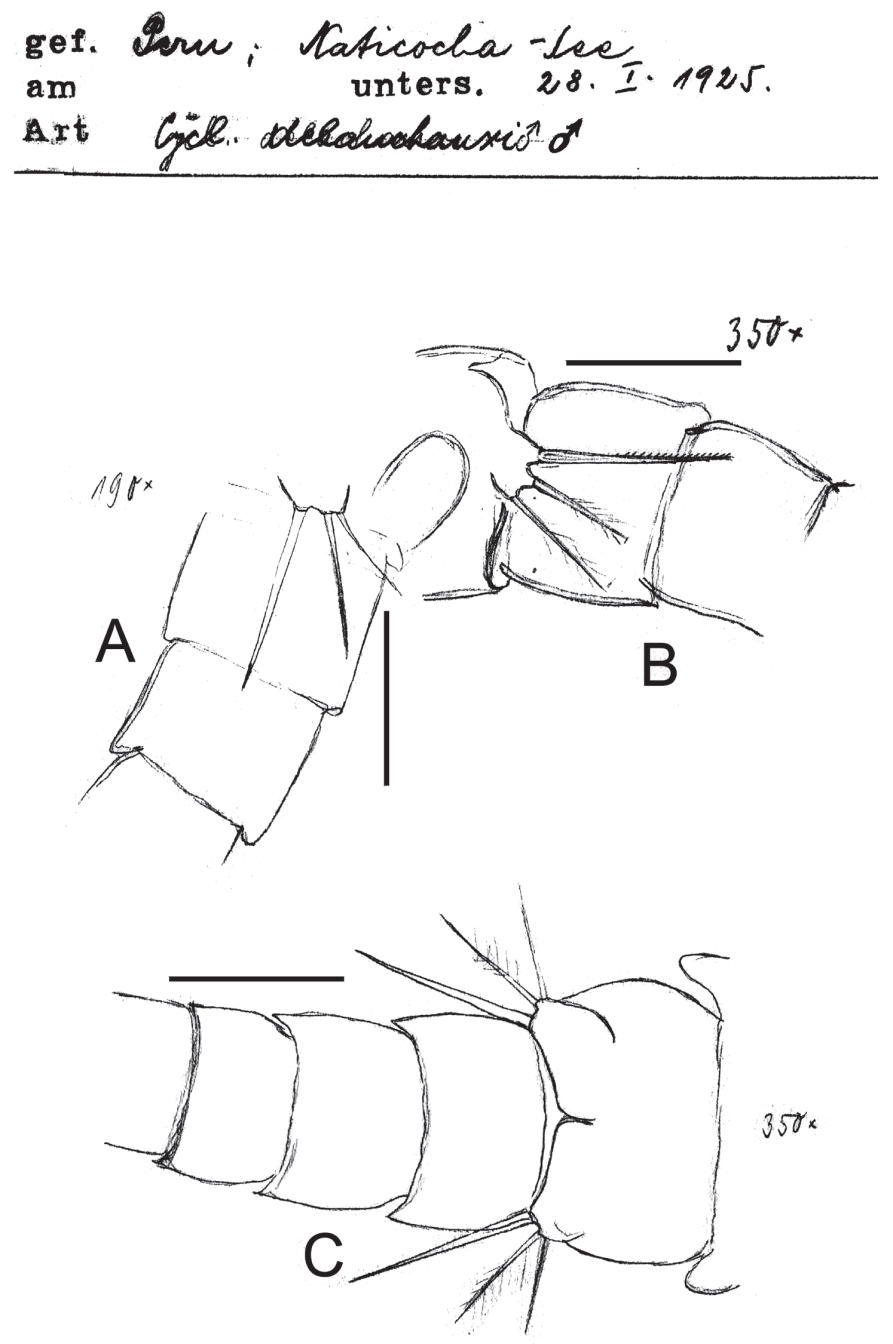
*Eucyclops delachauxi* (Kiefer, 1925). Original drawings of F. Kiefer. Male Paratype from Naticocha, Peru. **A–C** P6 and urosomites, ventral. Scale bars: **A–C** = 50 µm.

**Figure 5.**
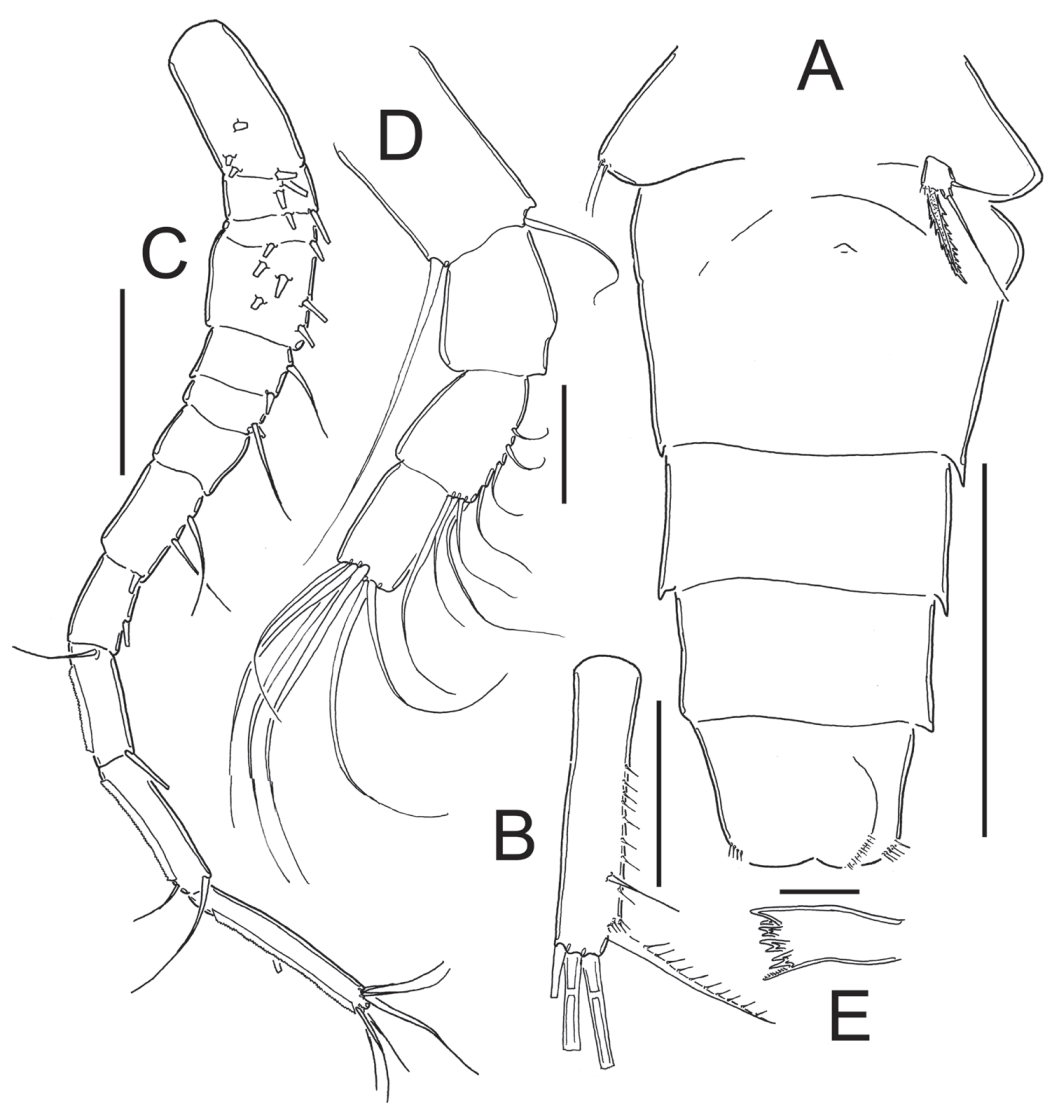
*Eucyclops delachauxi* (Kiefer, 1925). Author’s drawings. Female Holotype from Huaron, Peru. **A** Urosome, ventral view **B** Caudal ramus **C** Antennule **D** Antenna **E** Mandible (part). Scale bars: **A** = 100 µm; **B–C** = 50 µm; **D–E** = 20 µm.

a) *The female*: the general appearance as *Cyclops serrulatus*. Fifth segment of cephalothorax with lateral hair-setae. Last abdominal segment longer than the previous one. Caudal rami parallel, relatively short, about four times as long as wide; outer edge with *serra*, this is formed by a small number of (4–10) rather long, slender spinules. Inner edge naked (Fig. 1). Of the four setae on the end (apical), only the two median setae are strongly developed, the longest is approximately twice the length of the other, with fine plumage, almost the entire length is homogeneous, the innermost apical hair-like seta is as long as or slightly longer than the outermost seta, more similar to a spine. First antenna twelve–segmented, reaching only a little above of the posterior margin of the first segment of cephalothorax; the last three segments with a narrow hyaline membrane; the seta of the last member originates in the middle of the edge. The branches of all swimming legs with three segments. The terminal segment of the endopod of fourth leg is, usually, exactly twice as long as wide and its two setae on the inner margin and single seta on the outer margin are formed normally. Of the two apical spines, the innermost is strongly curved outside and it is longer than the segment, the outermost is just as long as the segment (Fig. 4). The rudimentary leg is a monomial plate; the inner edge is slightly distended, of the three elements the medial is significantly longer than the other two, which are approximately equal in length, the inner spine is, at its insertion, about twice as wide as one of the two setae (Fig. 2). The seminal receptacle was not correctly identified in the preserved animals (Fig. 1). Total length about 950 µm.

b) *The male*: slender and slightly smaller than the female. Last abdominal segment also noticeably longer than the previous one, its posterior margin provided on ventral side with only very few spinules, as well as in the female. Caudal rami parallel, also four times as long as wide. The *serra* is missing on the outer edge. Ratios and plumage of the two middle apical setae as in the female. Of the two short terminal setae in the male the inner seems to be always longer than the outer (Fig. 3). The final segment of the endopod of the fourth pair of legs with its setae and apical spines as in the female (Fig. 4); the fifth leg as well. The shape of the genital valve reinforcement is best seen in the figure (Fig. 5).

This *Cyclops*, of the numerous “*serrulatus*-like” that I know cannot be identified as one, it comes from two closely located lakes in the Andes of Peru (Huaron and Naticocha, 5140 m high) and has been collected by Ing. E. Godet in 1915. It is named after Dr. Th. Delachaux, Neuenburg, which I am indebted for the provision of his Cyclopoida material. The above description must be regarded as provisional. A more detail, equipped with longer pictures in the description of the *Cyclops*-forms from the mentioned lakes will be published in Archives of Hydrobiology.

**Description based on Kiefer’s material.**

**Material examined.** Holotype. Adult ♀ From Huaron, central highlands of Peru, specimen dissected (slide reference numbers SMNK00248, SMNK00249, SMNK00250). Additional material (adult ♂) from Lake Naticocha, Peru (slide reference number SMNK00253). Both Lake Huaron and Naticocha 5140 m high, samples collected by Ing. E. Godet in 1915. Deposited at the Staatliches Museum für Naturkunde Karlsruhe, Germany.

*Female*: Average length excluding caudal setae 950 µm. Five-segmented urosome (Figs 1A; 5A), relatively elongated; posterior margin of anal somite with one row of strong spinules. Genital double-somite symmetrical. Seminal receptacle typical of *serrulatus*-group, with rounded lateral arms. Genital double-somite 1.3 times as long as wide. Anal somite with a group of spinules at each side of anal opening, anal operculum rounded (see Fig. 2A). Length/width ratio of caudal ramus = 3.5–4.4; inner margin of caudal ramus naked. *Serra* with strong spinules covering 19.4–43% of outer margin, spinules about the same size (Figs 2B, 5B). Dorsal seta (VII) long: 0.6 times the length of caudal ramus and 0.9-1.0 times as long as outermost caudal seta (III). Ratio of innermost caudal seta (VI)/outermost caudal seta (III) = 1.1. Lateral caudal seta (II) inserted at 63–72% of caudal rami. All terminal caudal setae plumose.

*Antennule* (Figs 2C, 5C): 12-segmented. Armament per segment as follows (s = seta, ae = aesthetasc, sp = spine): **1**(5s), **2**(3s), 3(2s), **4**(**5s**), **5**(2s), 6 (1s+1sp), **7**(0s), 8(3s), **9**(2s), 10(2s), **11**(2s), 12(8s). Numbers in bold face indicate segments with incomplete ornamentation.

*Antenna* (Fig. 5D): Basis (2s + Exp), and 3-segmented Enp (1s, 8s and 6s). Basis ornamentation as follows (*sensu* Alekseev and Defaye 2011): N1(V), N2(4), N3(4), N4(6), N5(16), N6(6), N7(5), N8(3), N9(4), N10(3), N11(9), N12(6), N13(10), N14(3), N15(6), N16(2), N17(8).

*Labrum*, *Maxillule*, and *Maxilliped* not observable in the slides.

*Mandible* (Fig. 5E): with 6 tooth on gnathobase. Innermost margin with 1 spinulose seta.

*Maxilla* (Fig. 2D): Precoxa with row of small spinules on dorsal surface.

*P1-P4*: Endopod and exopods of all swimming legs 3-segmented. Armature formula as in Table 1.

**Table 1.** Setation formula of the swimming legs in the types (females and males) of the four *Eucyclops* species here studied; (spines in Roman numerals, setae in Arabic numerals). (–) represents structures not observed on the type material.

Species		Coxa	Basis	Exp	Enp
*Eucyclops delachauxi*	P1 P2 P3 P4	0-1 - 0-1 -	1-I - 1-0 -	I-1; I-1; III-5 -, -, - I-1; I-1; IV-5 -, -, -	0-1; 0-21-I-4 -, -, - 0-1; 0-2; 1-I-4 -, -, 1-II-2
*Eucyclops prionophorus*	P1 P2 P3 P4	0-1 - 0-1 0-1	1-I - 1-0 1-0	I-1, I-1, III-5 -, -, - I-1, I-1, IV-5 I-1, I-1, III-5	0-1, 0-2 1-I-4 -, -, - 0-1, 0-2, 1-I-4 0-1,0-2, 1-II-2
*Eucyclops bondi*	P1 P2 P3 P4	0-1 0-1 0-1 0-1	1-I 1-0 1-0 1-0	I-1, I-1, III-5 I-1, I-1, IV-5 I-1, I-1, IV-5 I-1, I-1, III-5	0-1, 0-2, 1-I-4 0-1, 0-2, 1-I-4 0-1, 0-2, 1-I-4 0-1, 0-2, 1-II-2
*Eucyclops leptacanthus*	P1 P2 P3 P4	0-1 0-1 0-1 -	1-I 1-0 1-0 -	I-1, I-1, III-5 I-1, I-1, IV-5 I-1, I-1, IV-5 -, -, -	0-1, 0-2, 1-I-4 0-1, 0-2, 1-I-4 0-1, 0-2, 1-I-4 -, -, 1-II-2

**Figure 6.**
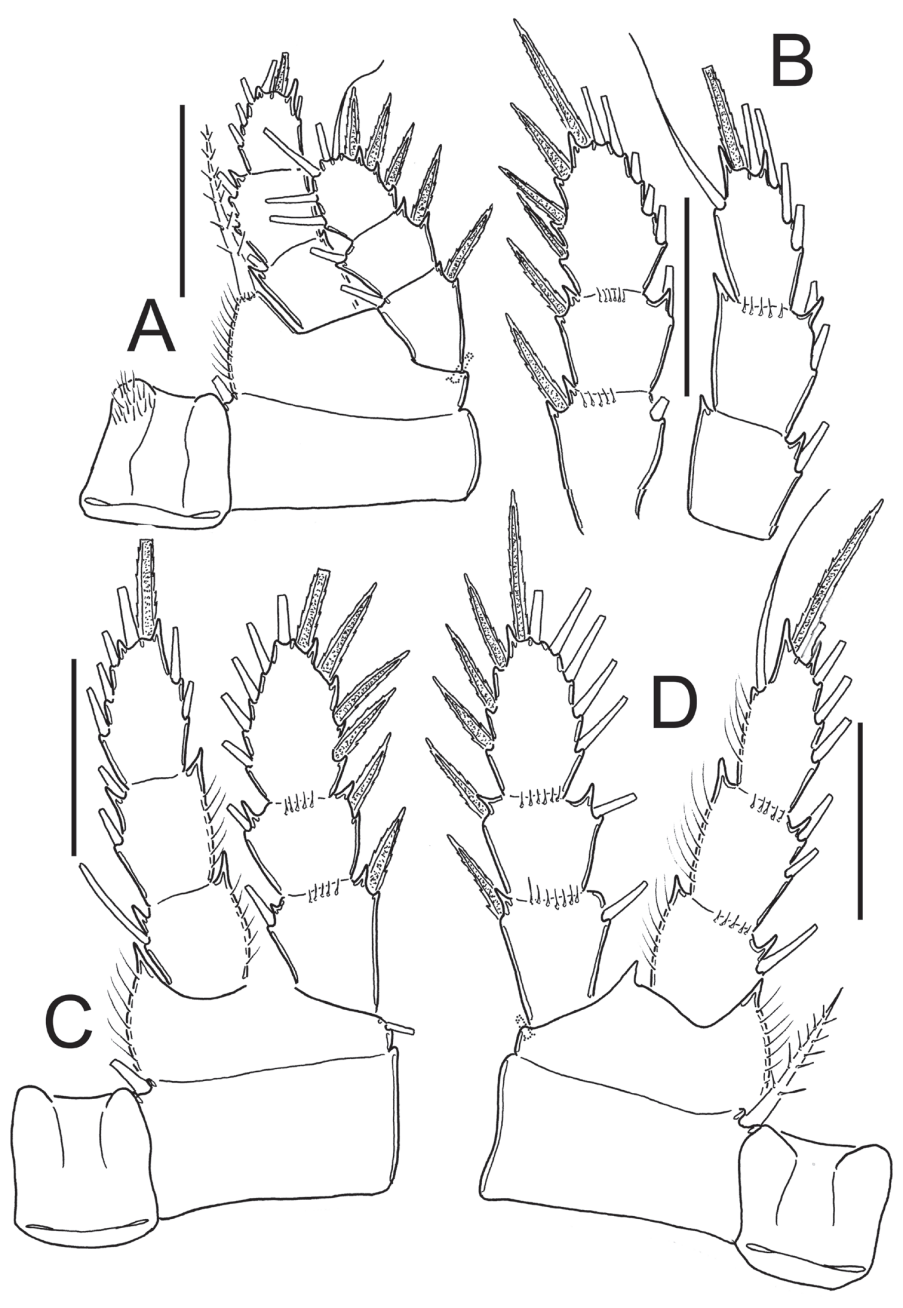
*Eucyclops delachauxi* (Kiefer, 1925). Author’s drawings. Female Holotype from Huaron, Peru. **A** P1 **B** P2 **C–D** P3. Scale bars: **A–D** = 50 µm.

*Leg 1* (Fig. 6A): Group of small hairs present in each side on anterior surface of intercoxal sclerite, distal margin with 2 rounded chitinized projections. Basipodal spine reaching middle length of Enp3, 0.8 times as long as Enp.

*Leg 2 and 3*: General shape as in Fig. 6B–D.

*Leg 4* (Fig. 3B): Intercoxal sclerite with short hairs in rows I, II, III. Coxopodite with row A, B (3-4), C+D (21), E (5), F and H (from Alekseev and Defaye 2011). Enp3P4: segment length/width ratio = 2.0-2.4; inner spine/outer spine = 1.1; inner spine /length of segment = 1.0; outer spine /length of segment = 0.9. Lateral seta of Enp3P4 inserted at 69% of the total length of the segment. Setae of Enp3P4 long and slender; lateral seta reaching beyond apical margin of inner spine (arrowed in Fig. 3B).

*Leg 5* (Fig. 1C): One free segment subrectangular, 1.4–1.8 times as long as wide, bearing 1 inner spine and 2 setae; medial seta about 1.7 times longer than outer seta. Inner spine 1.2–1.6 times longer than outer seta and 0.5–0.8 times as long as median seta. Inner spine 2.0–2.2 times as long as segment length.

*Leg 6* (Fig. 2E): Represented by small, flat plate with 1 slender and long seta and 2 small spines. Outer seta notably long, 12 times longer than medial spine and 6 times longer than inner spine.

**Figure 7.**
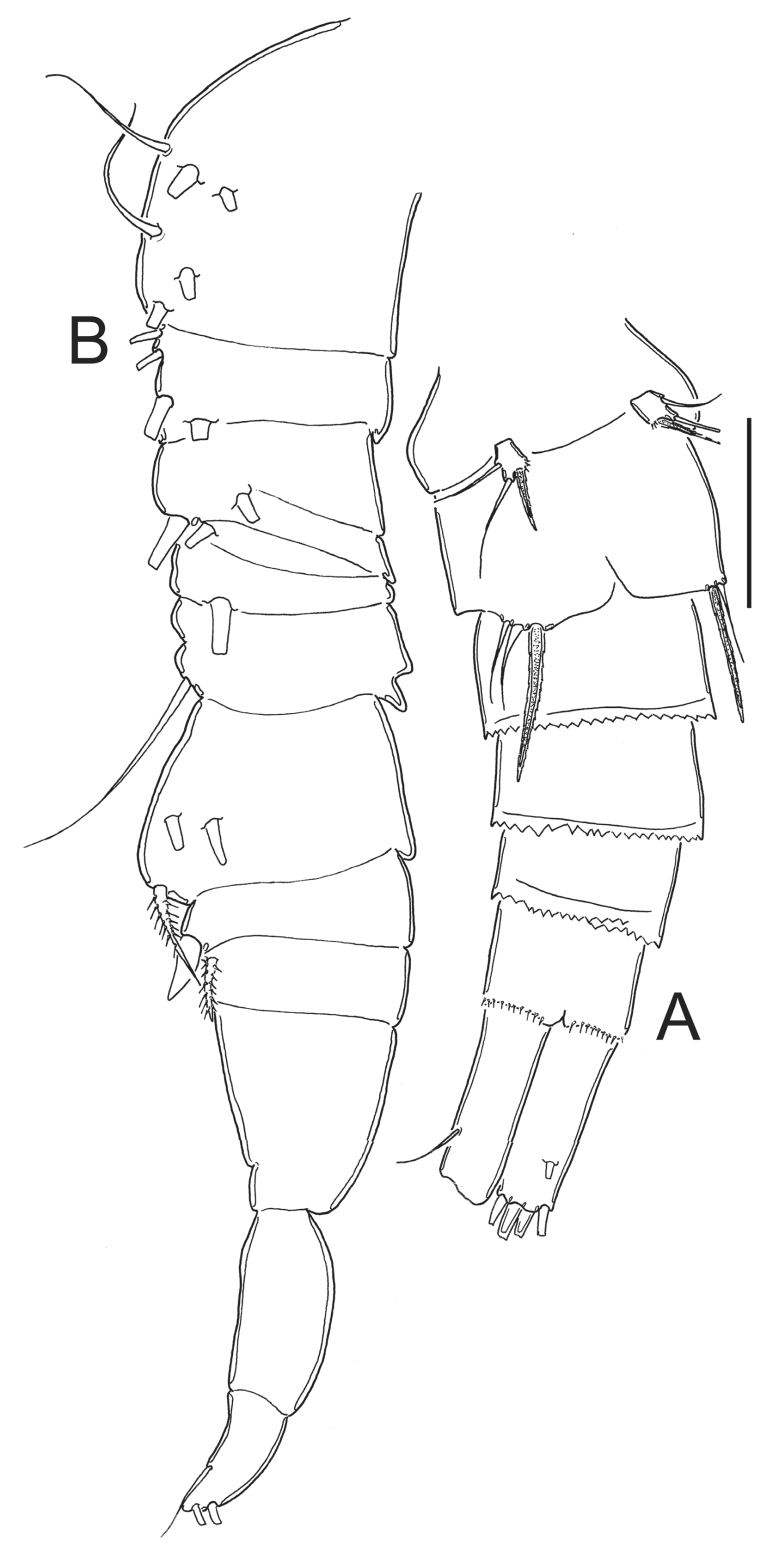
*Eucyclops delachauxi* (Kiefer, 1925). Author’s drawings. Male Paratype from Huaron, Peru. **A** Urosome, ventral view **B** Antennule. Scale bars: **A–B** = 50 µm.

*Male*: Urosome 6-segmented, posterior margin of urosomites serrated (Figs 4, 7A). Caudal ramus rectangular, 2.8–4.0 times as long as wide; inner margin of caudal ramus naked. Dorsal seta (VII) 0.6 times as long as caudal ramus, and 1.1 times as long as outermost caudal seta (III). Length ratio of innermost caudal seta (VI)/outermost caudal seta (III) = 1.2. Lateral caudal seta (II) inserted at 70% of caudal rami length. All terminal caudal setae plumose.

*Antennule* (Fig. 7B): ornamentation per segment incomplete to described in details.

*Leg 5* (Fig. 7A): segment subrectangular in shape, 1.5 times longer than wide, bearing 1 inner spine and 2 setae; medial seta about 1.4 times longer than outer seta. Inner spine 0.8 times longer than outer seta and 0.6 times as long as medial seta.

*Leg 6* (Figs 4, 7A): Represented by one small, flat plate placed near lateral margin of genital somite with 1 strong, long inner spine and 2 unequal setae. Inner spine reaching medial length of fourth urosomite. Inner spine about 1.3–1.8 times longer than medial seta and about 1.4–2.0 times longer than outer seta.

**Remarks.** In the publication posterior to the description of *Eucyclops delachauxi* made by Kiefer (1926) he pointed out the taxonomic problems within the genus derived from improperly weighted characters used for the species determinations. He encouraged the exploration and use of additional structures to achieve a more accurate definition of species in order to establish consistent patterns both taxonomically and biogeographically. Since its description, *Eucyclops delachauxi* has been recorded in Mexico, Colombia and Peru (Harding 1955; Gaviria 1994; Del Río and Valdivia 1989; Rodríguez-Almaráz 2000; Suárez-Morales 2004; Elías-Gutiérrez et al. 2008), but none of the records includes drawings or descriptions of the specimens that might allow us to compare them with the type material and confirm these records. In the original description, the length/width proportion of the Enp3P4 (2.0–2.4) was stated as a distinguishing character of this species but it is shared with other species (v. gr. *Eucyclops prionophorus*, *Eucyclops leptacanthus*, *Eucyclops bondi*, *Eucyclops pseudoensifer*) related to *Eucyclops delachauxi*, thus making it less informative to separate species. Another character remarked by Kiefer (1925) is the ornamentation of the outer margin of the caudal rami, which in comparison to other species is weakly ornamented, usually bearing 4–6 spinules; yet a significant variation has been observed, 7–8 (our observations) to sometimes 10 or 17 spinules. In a recent revision of the *Eucyclops serrulatus*-complex, Alekseev and Defaye (2011) mentioned another particular feature of this species, namely the relative length of the lateral seta on Enp3P4 in comparison to the length of the apical outer spine (lateral seta is as long as or even longer than the inner spine), a characteristic that is unique in the American representatives of *Eucyclops*. As mentioned by Kiefer, records of species should be consistent both taxonomically and geographically; hence, the records of *Eucyclops delachauxi* from Mexico of this probably South American species could be assignable to a different species. Until recently, *Eucyclops delachauxi* has been identified by less reliable characters, as described above. The observations included in Kiefer’s works (1925, 1926) about the ornamentation of caudal ramus were excluded by recent taxonomist and therefore many of the specimens recorded under the name of *Eucyclops delachauxi* could include records related to related species or even to species not yet described.

### *Eucyclops prionophorus* Kiefer, 1931

Figs 8–13

*Eucyclops* (s. str.) *prionophorus* Kiefer, 1931

*Eucyclops prionophorus*, Yeatman, in: Edmonson 1959, Smith and Fernando 1977, 1978, Harris 1978)

*Eucyclops prionophorus*, Einsle 1992

**Kiefer’s description.** *The female*: Caudal rami slender, four times as long as wide, row of spinules on the outer margin, distal spinules are longer and proximal spinules are slender (Fig. 2). First antenna twelve-segmented, slightly shorter than the cephalothorax, with a narrow hyaline membrane along the margin of last three segments. Spine formula of exopods of four swimming legs is 3443. Fifth leg with one segment, bearing 3 elements, of which the inner spine at its insertion is twice as wide as both other setae. Seminal receptacle is similar to that in *Eucyclops serrulatus*. Length excluding caudal setae 0.94 mm.

*The male*: Spine of genital plate (P6) extremely long, longer than the genital segment, with two short, slender, plumose setae. Body length excluding caudal setae 0.8 mm.

**Distribution.** North America, close to New Heaven.

**Description based on Kiefer’s material.**

**Material examined.** Holotype. Adult ♀ collected 05.05.1929 from New Haven, USA. Specimen dissected (slide number SMNK01508). Additional material from San Bernardino, Paraguay (slides numbers SMNK03103, SMNK03104). Staatliches Museum für Naturkunde Karlsruhe, Germany.

**Figure 8.**
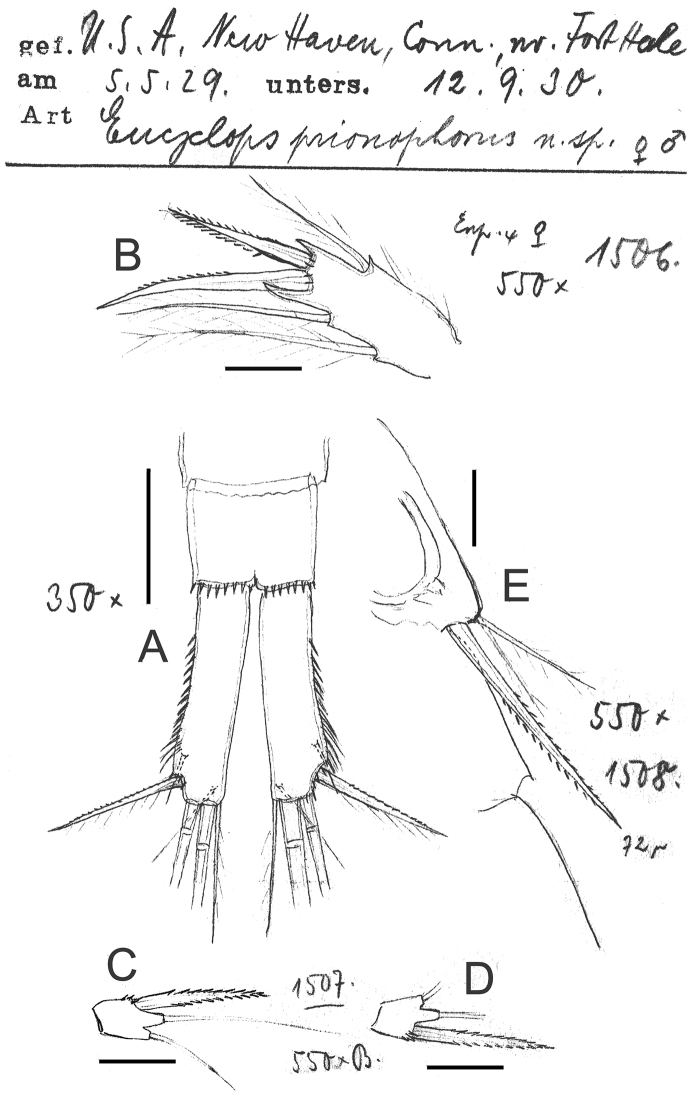
*Eucyclops prionophorus* Kiefer, 1931. Original drawings of F. Kiefer. **A** Female Holotype **B** female Paratype **A, C–E** from New Haven, U.S.A. **A** Caudal rami **B** Enp3P4 **C–D** P5 **E** P6. Scale bars: **A** = 50 µm; **B–E** = 20 µm.

**Figure 9.**
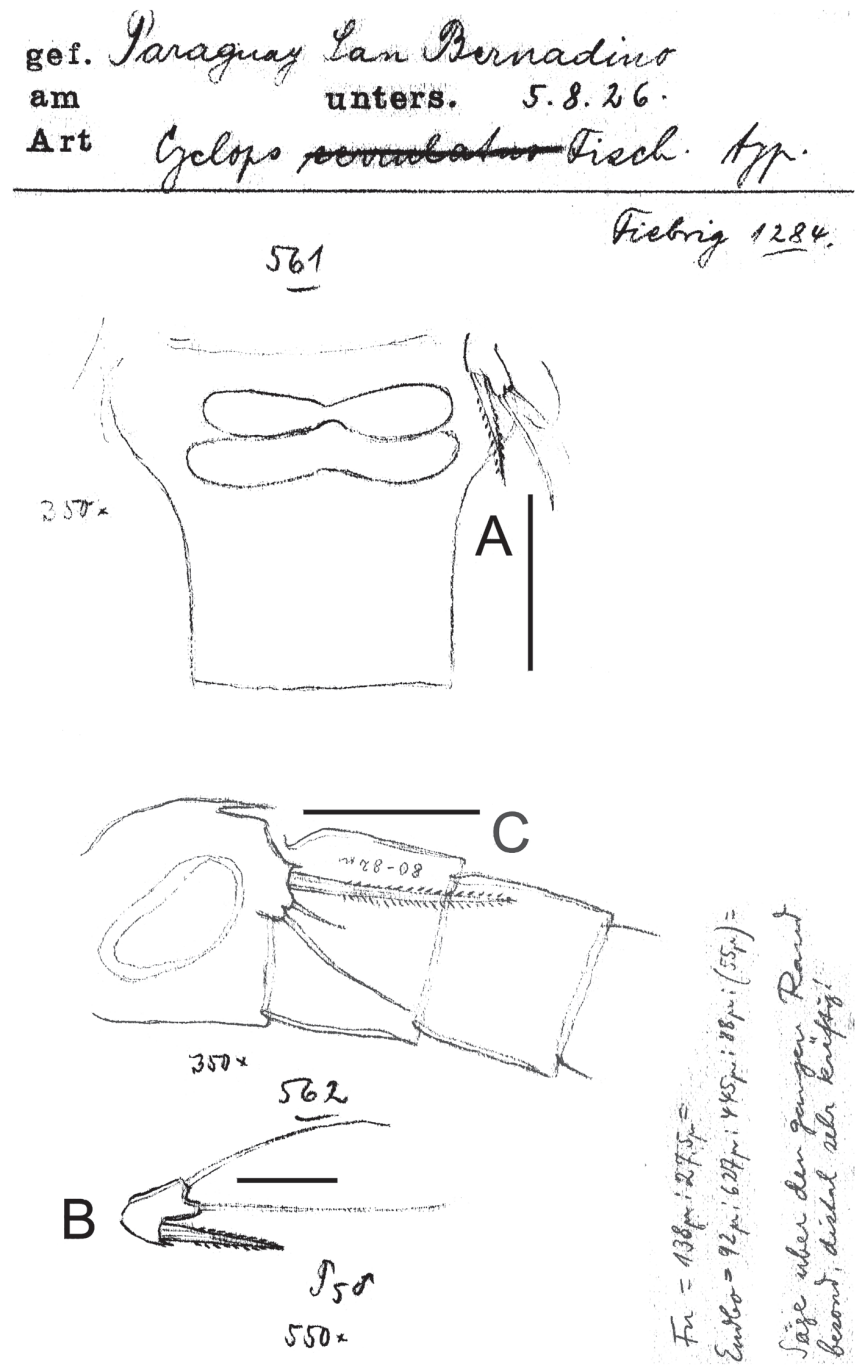
*Eucyclops prionophorus* Kiefer, 1931. Original drawings of F. Kiefer. Female **A** Male **B–C** from San Bernardino, Paraguay. **A** Genital double-somite and P5 **B** P5 **C** P6. Scale bars: **A, C** = 50 µm, **B** = 20 µm.

**Figure 10.**
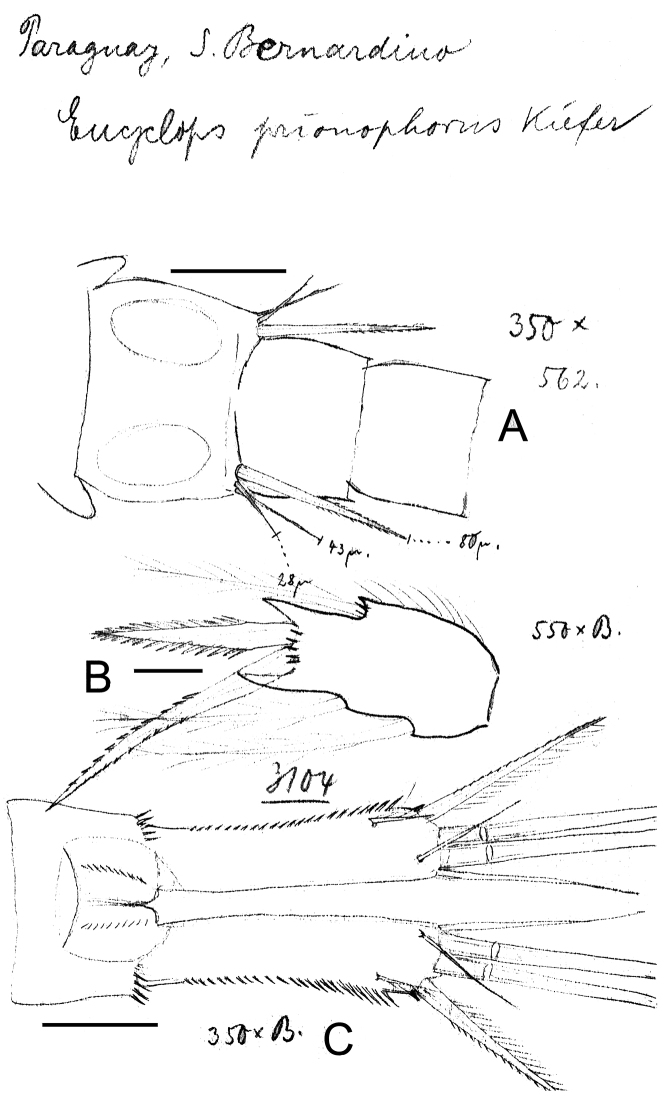
*Eucyclops prionophorus* Kiefer, 1931. Original drawings of F. Kiefer. Female **B–C** Male **A** from San Bernardino, Paraguay. **A** Genital somite and P6 **B** Enp3P4 **C** Anal somite and caudal rami. Scale bars: **A–C** = 50 µm.

**Figure 11.**
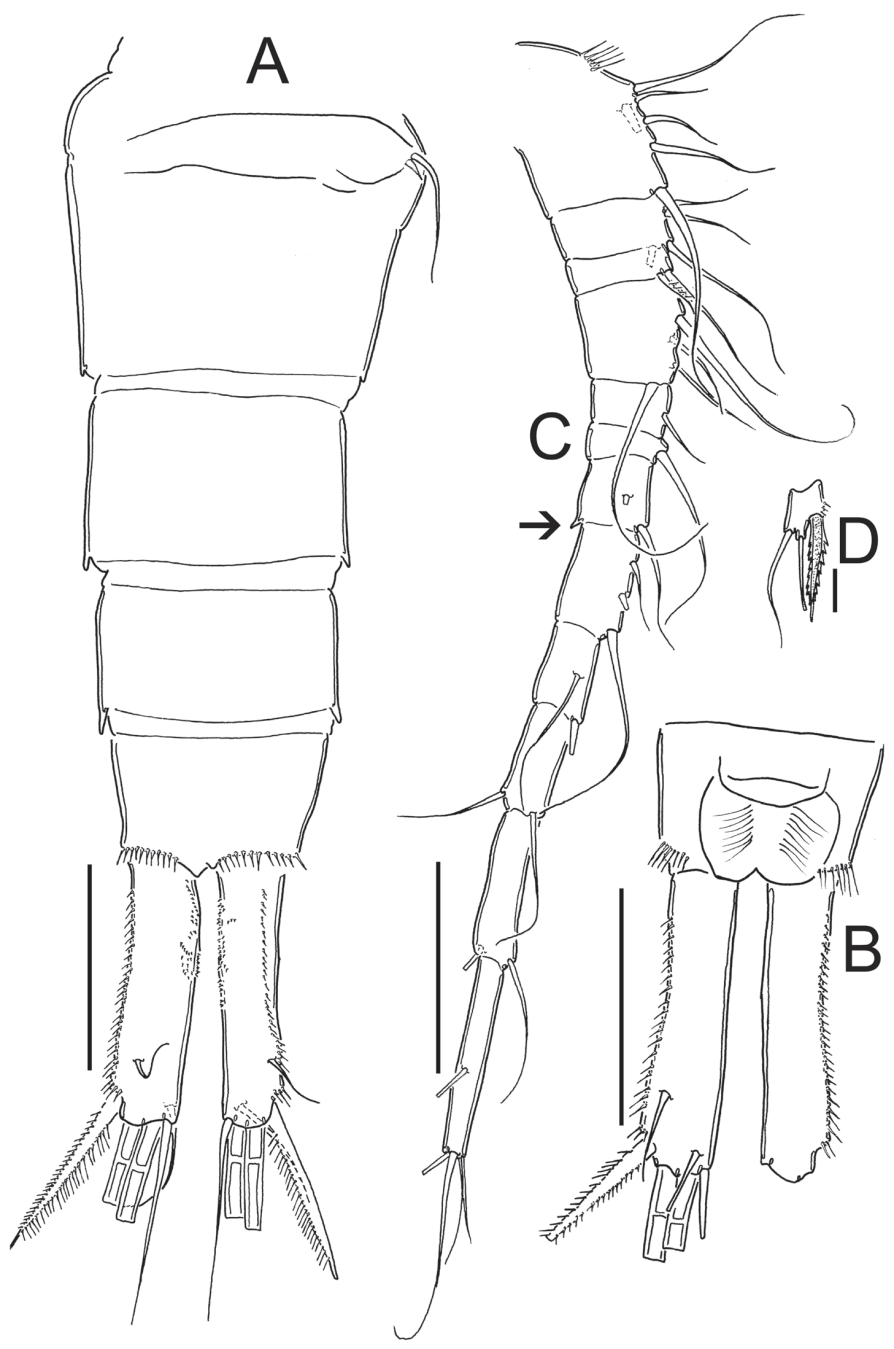
*Eucyclops prionophorus* Kiefer, 1931. Authors’ drawings. Female from San Bernardino, Paraguay. **A** Urosome, dorsal view **B** Anal somite and caudal rami **C** Antennule **D** P5. Scale bars: **A–C** = 100 µm, **D** = 20 µm.

*Female*: (Unless otherwise stated the character states are same in the holotype and in the Paraguay specimen) Average length excluding caudal setae 940 µm. Urosome 5-segmented (Fig. 11A), relatively elongated; urosomal fringes smooth or slightly serrated, posterior margin of anal somite with 1 row of relatively long spinules. Genital double-somite symmetrical (Fig. 9A). Seminal receptacle typical of the *serrulatus*-group, with rounded lateral arms on posterior margin. Genital double-somite slightly wider than long (about 1.1 times). Anal somite with one row of hair-like spinules in each side of anal opening, anal operculum slightly rounded (Figs 10C, 11B). Length/width ratio of caudal ramus = 4.0–4.5; inner margin of caudal ramus naked in specimens from New Haven and with groups of small spinules in specimens from San Bernardino (see Fig. 11A). Serra on outer margin with strong spinules covering 65–68% of lateral margin, spinules increasing in size distally (Figs 8A, 10C, 11B). Dorsal seta (VII) short, 0.4–0.6 times the length of caudal ramus, and 0.5–0.6 times as long as outermost caudal seta (III). Length ratio of innermost caudal seta (VI)/outermost caudal seta (III) =1.0–1.1. Lateral caudal seta (II) inserted at 75–77% of caudal ramus. All terminal caudal setae plumose.

*Antennule* (Fig. 11C): 12-segmented. Armament per segment as follows (s = seta, ae = aesthetasc, sp = spine): **1**(6s), 2(4s), 3(2s), **4**(4s), **5**(1s), **6** (1s), 7(2s, with small projection on inner margin, arrowed in Fig. 11C), 8(3s), **9**(2s), 10(2s), **11**(2s), **12**(5s). Numbers in bold indicate segments with incomplete ornamentation.

*Antenna*, *mouthparts and Leg 2*: not observable in slides.

*P1-P4*: Endopods and exopods of all swimming legs 3-segmented. Armature formula of all swimming legs as in Table 1.

**Figure 12.**
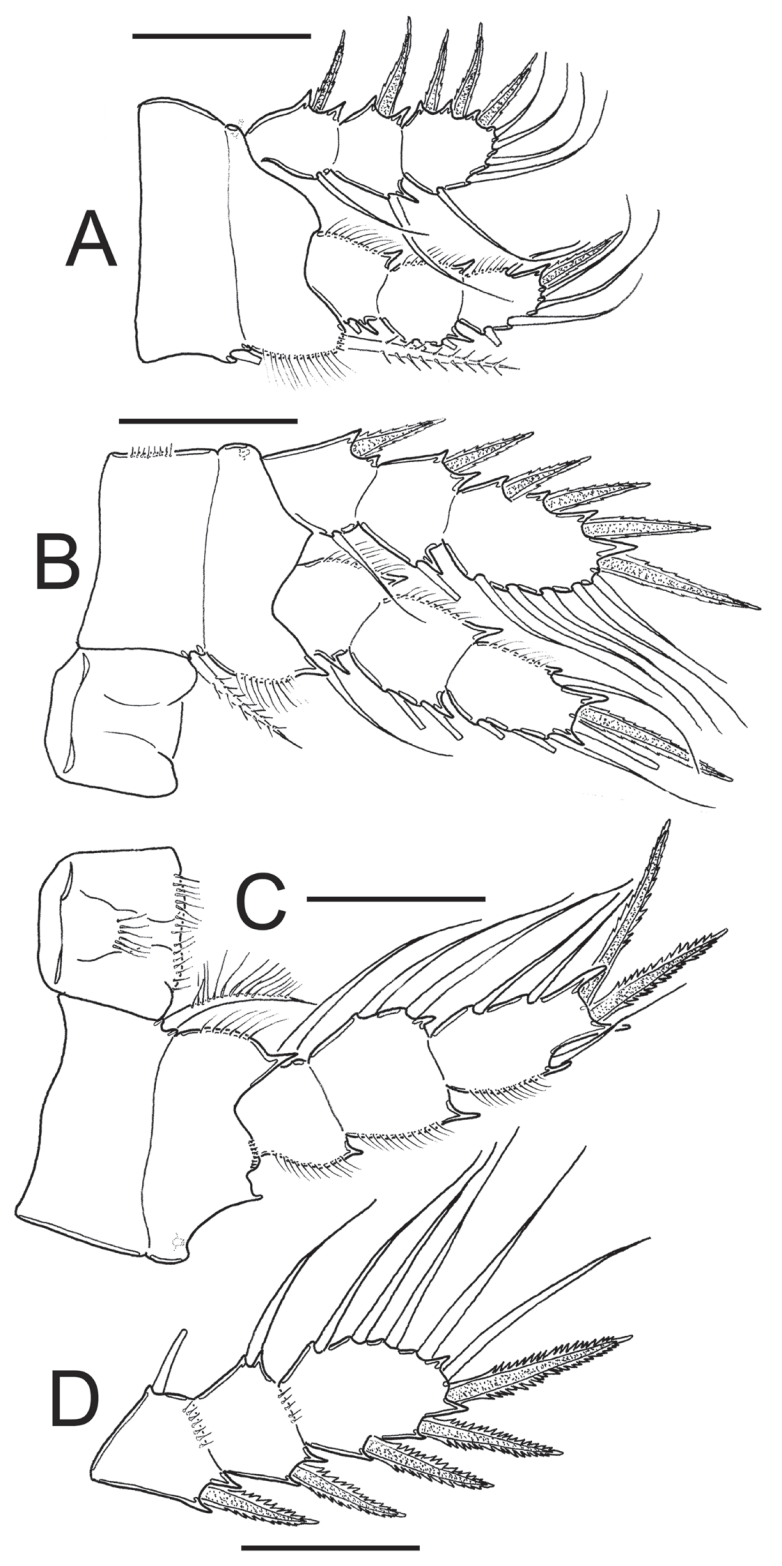
*Eucyclops prionophorus* Kiefer, 1931. Authors’ drawings. Female from San Bernardino, Paraguay. **A** P1 **B** P3 **C** P4 **D** Exp P4. Scale bars: **A–D** = 50 µm.

*Leg 1* (Fig. 12A): New Haven: Intercoxal sclerite without ornamentation and with 2 rounded chitinized projections. Coxa with strong biserially setulated inner coxal seta. Basipodal spine not reaching middle length of Enp 3; basipodal spine 0.6 times as long as total length of Enp. Third segment of Enp 1.5 times as long as wide, apical spine of Enp3 1.4 times longer than segment, apical most seta of Enp3 1.2 times longer than apical spine. Spines of Exp slightly elongated. San Bernardino: intercoxal sclerite not available. Basipodal spine reaching beyond middle length of Enp3; basipodal spine 0.75 times as long as total length of Enp. Third segment of Enp 1.2 times as long as wide, apical spine of Enp3 1.2 times longer than segment; apicalmost seta of Enp3 1.6 times longer than apical spine.

*Leg 3* (Fig. 12B): No ornamentation observed on intercoxal sclerite, distal margin with 2 rounded projections. Coxa with strong, biserially setulated inner coxal seta. Coxa with row of tiny spinules along outer margin. Enp3 1.7–1.8 times as long as wide, apical spine on Enp3 1.2 times as long as segment, Exp3 1.5–1.7 times as long as wide, apicalmost spine of Exp3 1.1 times as long as segment.

*Leg 4* (Fig. 12C–D): Intercoxal sclerite with rows I, II and III. Row I with 7 long spinules in each side and a small gap between. Row II with 6 spinules on middle margin. Row III divided in 3 sections, first one with 3 short spinules, middle section with 2 short spinules and third section with 2–3 short spinules (all observed in San Bernardino’s specimens). Caudal coxal surface with spinule formula: A, B (4), C+D (6) (*sensu* Alekseev and Defaye 2011). Coxal spine with heteronomous setulation: with long hairs basally, and spinules distally; lateral edge of coxal spine with 3 spinules apically, proximal part naked. Enp3P4: segment length/width ratio = 2.0–2.5; inner/outer spines = 1.1–1.5; inner spine/segment length = 1.0–1.5; outer spine/segment length = 0.8–1.1. Lateral seta of Enp3P4 inserted at 60–70% of the total length of segment. Modified setae present on Enp3P4 in specimens from Paraguay. Enp3 setae long in specimens from New Haven and Paraguay. Exp3 1.6–1.8 times as long as wide, apicalmost spines of Exp3 0.9–1.2 times as long as segment.

*Leg 5* (Figs 8C–D, 9A, 11D): Free segment subrectangular, 1.4–1.8 times longer than wide, bearing 1 inner spine and 2 setae; median seta longer than outer seta (1.0–1.7 times) and 1.3–1.6 times times longer than inner spine. Inner spine 1.7–2.0 times as long as segment.

*Male*: Average length excluding caudal setae 800 µm. Urosome 6-segmented, posterior margin of urosomites smooth. Caudal ramus 3.5 times as long as wide, inner margin naked. with a group of spinules present at insertion of lateral seta. Ratio of innermost caudal seta (VI)/outermost caudal seta (III) = 1.6. All terminal caudal setae plumose.

**Figure 13.**
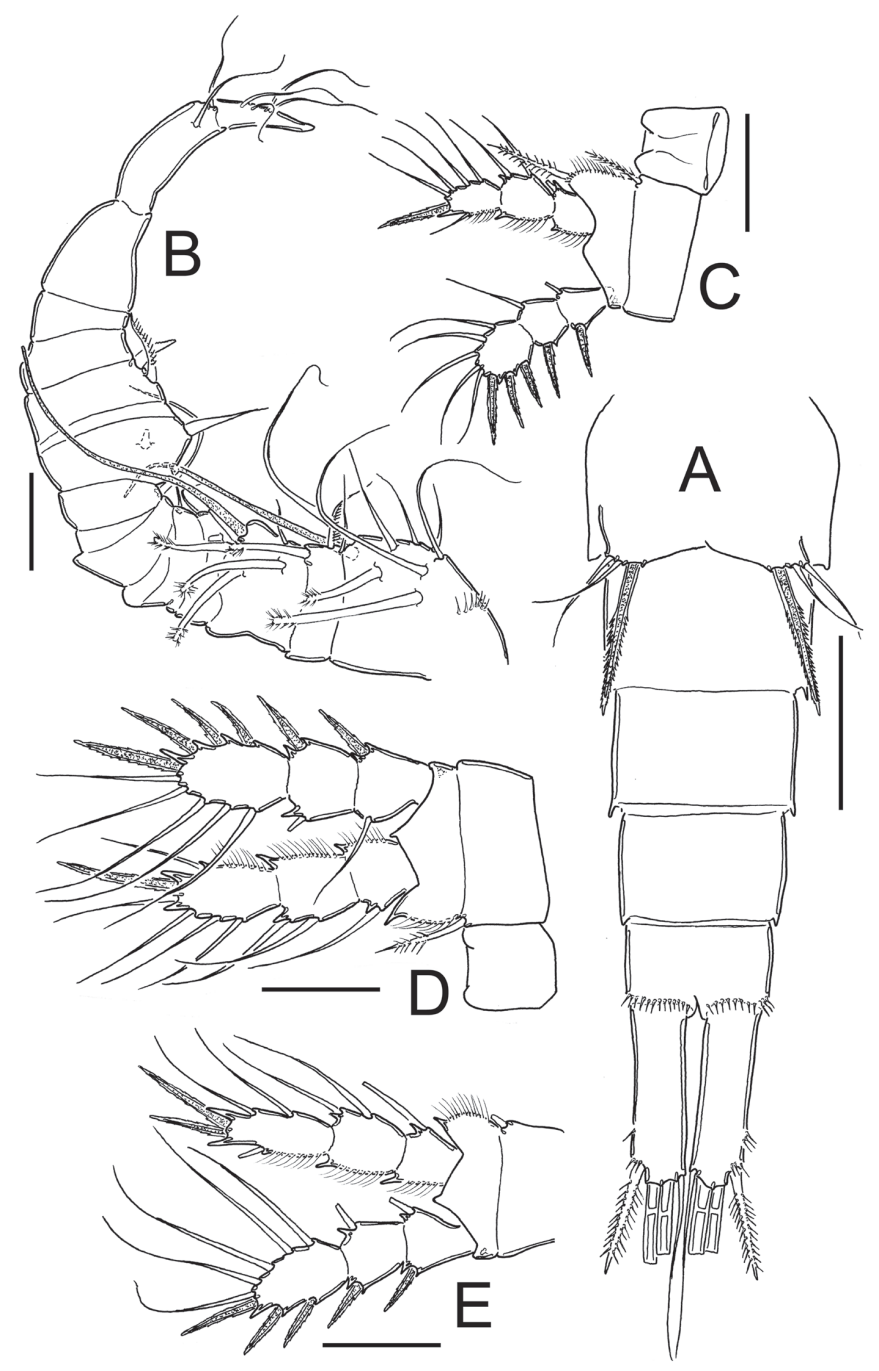
*Eucyclops prionophorus* Kiefer, 1931. Authors’ drawings. Male from San Bernardino, Paraguay. **A** Urosome, ventral view **B** Antennule **C** P1 **D** P3 **E** P4. Scale bars: **A** = 100 µm, **B–E** = 50 µm.

*Antennule*: 1-segmented (Fig. 13B), armament per segment as follows (s = seta, modified seta = ms, ae = aesthetasc, sp = spine): 1(6s + 2ms + 1ae); 2(42 + 1ms); 3(2s+3ms +1ae); 4(1s); 5(1s); 6(0); 7(1s); 8(2s); 9(1s +1sp); 10(0); 11(0); 12(0); 13(2s); 14 (4s).

*Leg 5*: Free segment subrectangular, 1.4 times longer than wide, bearing 1 inner spine and 2 setae; median seta longer than outer seta (about 1.3 times).

Inner spine 0.6 times longer than outer seta and 0.4 times as long as median seta.

*Leg 6* (Figs 8E, 9C, 10A, 13A): Represented by small, low plate near lateral margin of genital somite, armed with 1 strong and long inner spine and 2 unequal setae. Inner spine not reaching half length of fourth urosomite. Inner spine about 1.6–2.5 times longer than median seta and 1.2–1.8 times longer than outer seta.

**Remarks.** Since its description in 1931 from a material collected in the USA, *Eucyclops prionophorus* has been recorded from various habitats in the Americas including the Laurentian Great Lakes in Canada and the USA, the Chihuahuan Desert in Mexico, and water bodies in savannahs and deciduous forests in Argentina, Paraguay and Uruguay (Kiefer 1936; Czaika 1974, 1978; Robertson and Gannon 1981; Dussart and Fernando 1990; Einsle 1992; Reid and Marten 1995; Suárez-Morales and Reid 1998; Grimaldo-Ortega et al. 1998; Suárez-Morales 2004; Suárez-Morales et al. 2010; Mercado-Salas and Suárez-Morales 2012). In the description made by Kiefer (1931), the key characters of the species include the ornamentation on the outer margin of the caudal rami (spinules distally increasing in size), the remarkably strong (wide) spine of the fifth leg, and the extremely long spine on the sixth leg of the male. After Kiefer’s contribution (1931), the most complete comparisons among populations of *Eucyclops prionophorus* were provided by Einsle (1992) based on American material from Kiefer’s collection. In this paper, Einsle stated that the type material of the species was damaged and therefore it couldn’t be used for the redescription of the species. Hence, he used the material from Paraguay identified by Kiefer as *Eucyclops prionophorus* to point out the main characteristics of the species, as follows: 1) dorsal caudal seta shorter than innermost and outermost caudal setae and shorter than caudal ramus; 2) basipodal seta of P1 reaching middle of Enp3P1;) setae of exopodites of P3 and P4 transformed, spatulate; 4) setae on Enp3 P4 short (differing from Kiefer’s description) and; 5) the outer edge of P5 wider and longer than the central lobe. Our own observations on Kiefer’s material from Paraguay, revealed that the main characteristic of *Eucyclops prionophorus* is the short dorsal caudal seta length being shorter than in any other closely related species (e.g. *Eucyclops bondi* and *Eucyclops conrowae*), but not as short as in *Eucyclops pseudoensifer* (Dussart 1984; Suárez-Morales and Walsh 2009). A remarkable feature found in the material from Paraguay is the ornamentation of the inner margin of the caudal rami, where we observed a group of tiny spinules that was never reported for this species (Fig. 11A). This character should be compared in other populations as well, in order to verify its diagnostic value: whether it is simple intraspecific variation or a unique species-specific character. Another structure that could be useful to distinguish this species from its congeners is the ornamentation of the intercoxal sclerite of the fourth leg: in *Eucyclops prionophorus* row I includes long and slender spinules (Fig. 12C), while in *Eucyclops bondi* and *Eucyclops conrowae* this row always consists of small and strong spinules, and in *Eucyclops pseudoensifer* row I consists of very long hairs. In our observations of the specimens identified as *Eucyclops prionophorus* from Mexico, we found a possible pattern in the ornamentation of the intercoxal sclerites of legs 3 and 4; in all the specimens possessing strong spinules in row I of P4, the intercoxal sclerite ornamentation of P3 also includes spinules, at least in one of the three rows of the plate. In case of the individuals possessing long hairs in row I of P4, the three rows of the P3 intercoxal sclerite always consist of long hairs. These observations will be discussed and compared in another manuscript about the Mexican fauna of *Eucyclops*. Records of *Eucyclops prionophorus* in the Americas appear to be well determined, at least those which include drawings of the caudal rami and the fourth leg, showing the characteristics remarked by Kiefer (1931, 1936) and Einsle (1992). Here we also present the first illustration of the male antennule (Fig. 13B) of this species; we found modified setae on segments 1, 2 and 3 and aesthetascs on segments 1 and 3; this pattern differs from the presented by Alekseev et al. (2006) for *Eucyclops serrulatus*, in which aesthetascs are reported only on segments 2, 3, 4, 6 and 10.

### *Eucyclops bondi* Kiefer, 1934

Figs 14–17

*Eucyclops* (s. str) *Bondi* Kiefer, 1934

*Eucyclops* (s. str.) *Bondi* Kiefer, 1936

*Eucyclops bondi*, Smith and Fernando 1980; Reid 1992

**Kiefer’s description.**

*Female*: General aspect as the American *Eucyclops prionophorus*. Caudal rami 3.5 times longer than wide, with rami slightly divergent. Inner margin of caudal ramus naked, outer margin strongly ornamented with strong spinules. Proximal spines small but distal spines long (Fig. 4). Innermost apical seta longer than outermost seta; two middle setae show strongly heteronomous plumage.

Antennule only slightly longer than cephalothorax, bearing 12 segments, last three segments with a narrow hyaline membrane along margin.

Swimming legs normally segmented, with spines and setae. Third endopod of P4 two times longer than wide, inner spine longer than segment and even 1.5 times longer than the outer spine. The connecting plate of this leg is hairy on the free margin. Rudimentary leg (P5) with a slender inner spine, inner spine is at its insertion about twice as wide as one of the both setae. Seminal receptacle without special characteristics. Total length of animals, excluding apical setae of caudal ramus, 720–800 µm.

*Male*: total length, excluding apical setae of caudal ramus, 580–600 µm. As a main characteristic the reinforcement of genital somite (P6) should be considered. Of the three elements, the inner spine only measures 22–23 µm, clearly differing from the similar North American species *Eucyclops prionophorus*, the outermost plumose seta is longer than the spine, the median seta is as long as the spine. In the male of *Eucyclops prionophorus* the inner spine is more than three times longer, 71 µm.

**Description based on Kiefer’s material.**

**Material examined.** Holotype. Adult ♀ collected 16.02.1933 from Trou Caiman, Haiti, specimen dissected (slide reference numbers SMNK02079, SMNK02080). Additional material from Laguna Rincon, Haiti (slide reference numbers SMNK02393, SMNK02394). Staatliches Museum für Naturkunde Karlsruhe, Germany.

**Figure 14.**
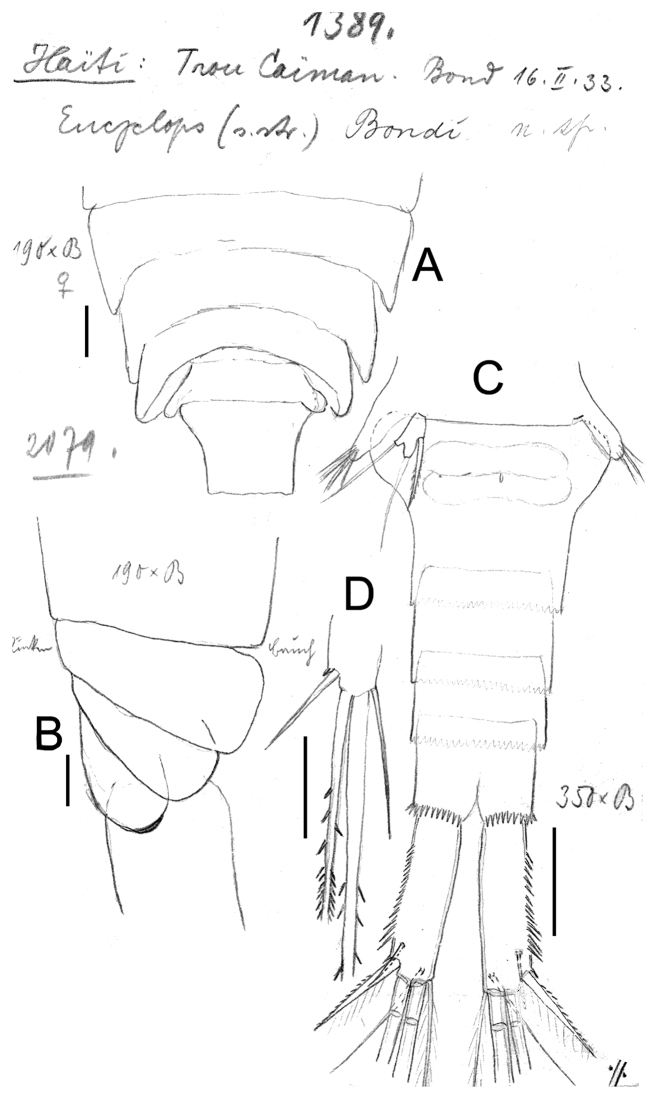
*Eucyclops bondi* Kiefer, 1934. Original drawings of F. Kiefer. Female Holotype from Trou Caiman, Haiti. **A** Prosome 2–5 and genital somite, dorsal view **B** Prosome 2–5, lateral view **C** Urosome, ventral view **D** Caudal setae of CR. Scale bars: **A–D** = 100 µm.

*Female*: Body length excluding caudal setae 720–800 µm. Prosome expanded at first and second somite, symmetrical in dorsal view. Urosome 5-segmented (Figs 14C, 16A–B), relatively elongate. Urosomal fringes strongly serrated, posterior margin of anal somite with row of long spinules. Genital double-somite symmetrical. Seminal receptacle typical of *serrulatus*-group, with rounded lateral arms on posterior margin. Genital double-somite 1.1 times as long as wide. Anal somite with hair-like spinules in anal opening, anal operculum slightly rounded (Fig. 16A). Length/width ratio of caudal ramus = 3.5. Inner margin of caudal ramus naked, outer margin partially covered (53–57%) by strong spinules which increase in size distally (Figs 14C, 16B). Dorsal seta (VII) long: 0.8 times of caudal ramus length, and 1.26–1.4 times as long as outermost caudal seta (III). Length ratio of innermost caudal seta (VI)/outermost caudal seta (III) = 1.07–1.25. Lateral caudal seta (II) inserted at 77–80% of caudal rami. All terminal caudal setae plumose.

**Figure 15.**
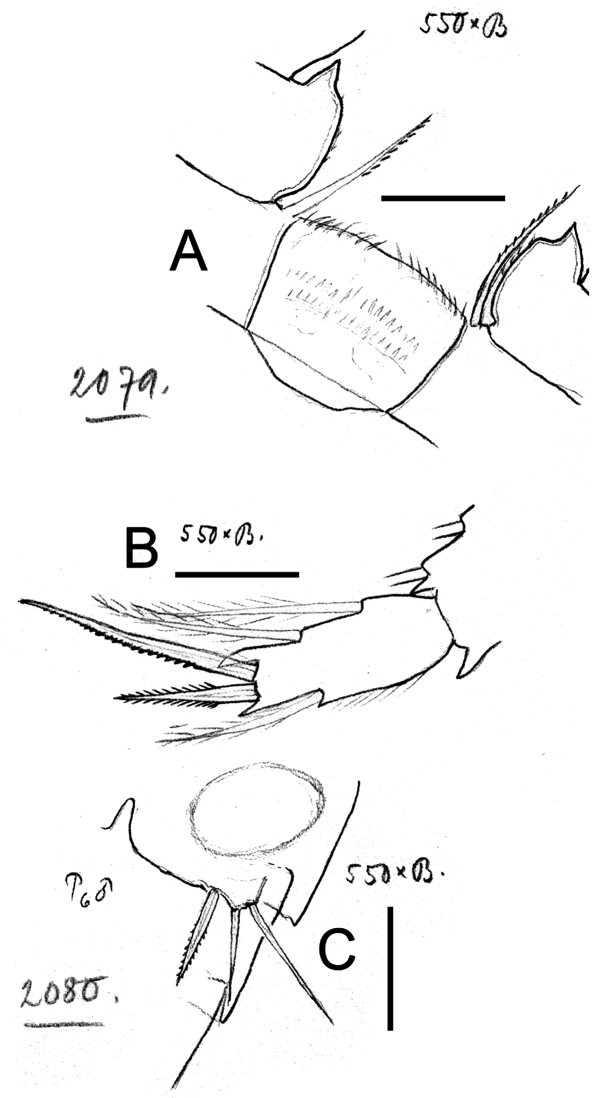
*Eucyclops bondi* Kiefer, 1934. Original drawings of F. Kiefer. Female Holotype **A–B** and male Paratype **C** from Trou Caiman, Haiti. **A** Intercoxal sclerite and coxal spines P4 **B** Enp3P4 **C** P6. Scale bars: **A–C** = 50 µm.

**Figure 16.**
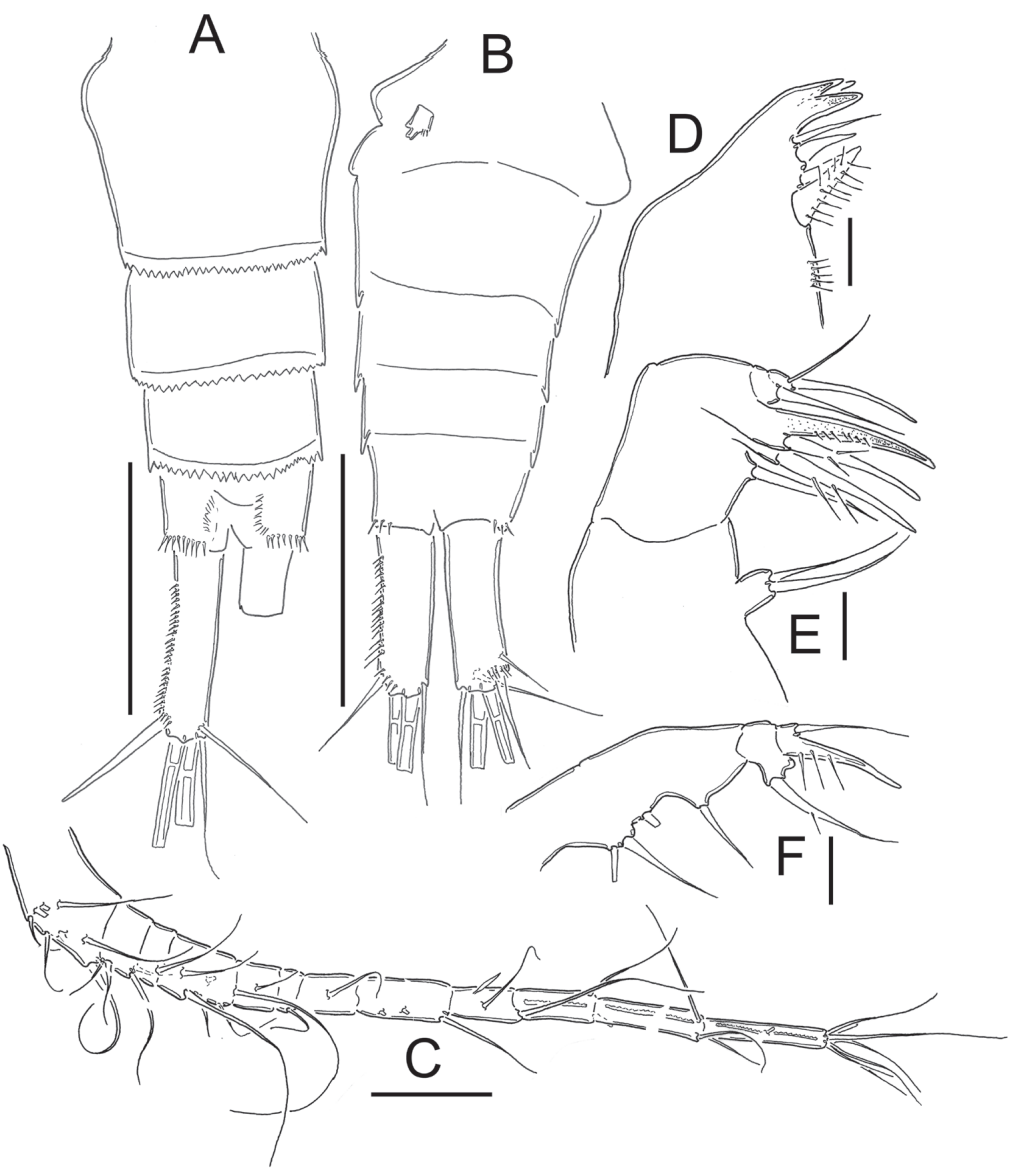
*Eucyclops bondi* Kiefer, 1934. Authors’ drawings. Female Holotype from Trou Caiman, Haiti. **A** Urosome, dorsal view **B** Urosome, ventral view **C** Antennule **D** Maxillule **E** Maxilla **F** Maxilliped. Scale bars: **A–B** = 100 µm; **C** = 50 µm; **D–F** = 20 µm.

*Antennule* (Fig. 16C): 12-segmented, reaching second prosomite; three distal segments with narrow hyaline membrane. Armament per segment as follows (s = seta, ae = aesthetasc, sp = spine): 1(8s), 2(4s), **3**(1s), 4(6s), **5**(1s), 6 (1s+1sp), **7**(1s), 8(3s), 9(2s+1ae), 10(2s), 11(3s), 12(8s). Numbers in bold indicate segments with incomplete ornamentation. Aesthetasc of ninth segment short, reaching posterior margin of segment.

*Antenna*, *Labrum* and *Mandible*: not observable in slides.

*Maxillule* (Fig. 16D): precoxal arthrite with naked surface, with 3 strong chitinized distal claws. Spiniform seta on frontal side and palp not observed.

*Maxilla* (Fig. 16E): praecoxa and coxa partially fused. Praecoxa with 2 armed setae on endite. Coxal surface naked, bearing 1 biserially plumose seta. Distal endite of coxopodite well developed, with 2 apical setae, 1 strong and furnished with spinules and the other noticeably thicker and longer. Basal claw of basis with proximal row of spinules and 1 chitinized armed seta. Endopod 2-segmented, first segment with 1 seta, second with 2 setae.

*Maxilliped* (Fig. 16F): syncoxa naked, bearing 3 setae. Maxillipedal basis with 1 seta and no observable additional ornamentation. Endopod 2-segmented: Enp1 with 1 long, strong seta, Enp2 with 2 setae, proximal 1 chitinized and fused with segment, apical seta normal.

*P1–P4*: Endopod and exopods of all swimming legs three-segmented. Armature formula of all swimming legs as in Table 1.

**Figure 17.**
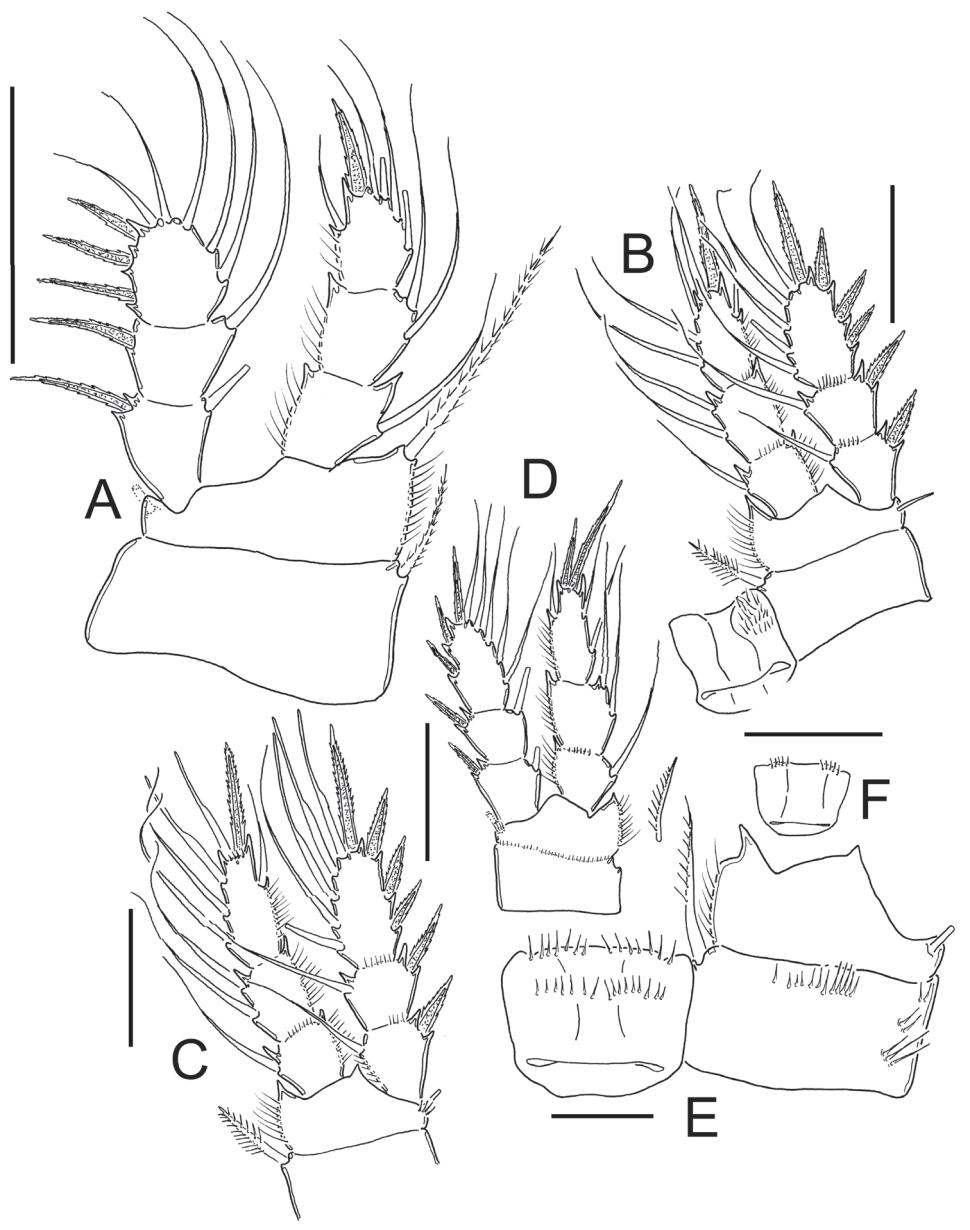
*Eucyclops bondi* Kiefer, 1934. Authors’ drawings. Female Holotype from Trou Caiman, Haiti. **A** P1 **B** P2 **C** P3 **D** P4 **E** P4, caudal surface **F** Intercoxal sclerite P4, frontal surface. Scale bars: **A–D, F** = 50 µm, **E** = 20 µm.

*Leg 1* (Fig. 17A): Coxa with strong, biserially setulated inner coxal seta. Basipodal spine long, reaching apical margin of Enp3; basipodal spine as long as endopod. Third endopodal segment 1.5 times as long as wide, apical spine of Enp3 0.9 times longer than length of Enp3, apicalmost seta of Enp3 1.4 times longer than apical spine. Spines of all exopodal segments elongate.

*Leg 2* (Fig. 17B): Single group of small spinules in each side on anterior surface of intercoxal sclerite. Distal margin of intercoxal sclerite with 2 round chitinized projections. Coxa with strong, biserially setulated inner coxal seta. Enp3 1.9 times as long as wide, apical spine 1.2 times longer than length of Enp3, apicalmost seta of Enp3 1.4 times longer than apical spine.

*Leg 3* (Fig. 17C): Coxa with strong biserially setulated coxal seta. Enp3 2.2 times longer than wide, apical spine as long as Enp3, apicalmost seta of Enp3 as long as apical spine. Enp3 and Exp3 with modified setae on.

*Leg 4* (Figs 15A–B, 17D–F): Intercoxal sclerite with rows I, II and III. Row I with 7 strong spinules in each side and small gap in between. Row II with 16–18 spinules, row III with 14 strong spinules. Caudal coxal surface with spinule formula as: A, B (3), C+D (12), E (2), F, G (2), H, I. Inner spine of coxa with heteronomous setulation: basally with long hair-like setules, distally with spine-like setules; lateral edge of coxal spine naked. Enp3P4 length/width ratio 2.5; length ratio inner/outer spines of Enp3P4 = 1.6–1.8; length ratio inner spine in Enp3P4/Enp3P4 = 1.3; length ratio outer spines in Enp3P4/Enp3P4 = 0.7. Lateral seta in Enp3P4 inserted at 71% of segment length. Enp3 and Exp3 with normal setae.

*Leg 5* (Fig. 14C): Free segment subrectangular, 2 times longer than wide, with 1 inner spine and 2 setae; median seta as long as outer seta (1:1) but about 1.3 times longer than inner spine. Inner spine 1.6 times as long as segment.

*Male*: Body length excluding caudal setae 580–600 µm. Urosome 6-segmented, posterior margins of urosomites smooth. Caudal rami 2.6 times longer than wide; medial margin of caudal ramus naked, strong spines at insertion point of lateral caudal seta (II).

*Leg 6* (Fig. 15C): Represented by small, flat plate near lateral margin of genital double somite with 1 strong short inner spine and 2 unequal setae. Inner spine not reaching posterior margin of third urosomite. Inner spine about 0.7 times as long as outer seta and as long as median seta.

**Remarks.** After Kiefer’s description (1934) of *Eucyclops bondi*, this species has been recorded from Colombia, Costa Rica, Cuba, Dominican Republic, Guatemala, Haiti, Mexico, Trinidad, and the USA (Florida) (Collado et al. 1984; Reid 1992; Suárez-Morales et al. 1996; Suárez-Morales and Reid 1998; Grimaldo-Ortega et al. 1998; Bruno et al. 2005; Dussart and Defaye 2006; Gaviria and Aranguren 2007; Elías-Gutiérrez et al. 2008; Mercado-Salas 2009; Suárez-Morales et al. 2010; Suárez-Morales and Walsh 2009; Mercado-Salas et al. 2012; Mercado-Salas and Suárez-Morales 2012). There are only a few records that include drawings of the main structures used in the identification of this species, thus allowing us to make some comparisons and speculate about their status. The records of *Eucyclops bondi* from Costa Rica made by Collado et al. (1984) included drawings of both female and male specimens, and by comparing both sexes we conclude that the Costa-Rican records are not assignable to *Eucyclops bondi*. One of the main characteristics mentioned in Kiefer’s description of this species is the particular shape and length of the three elements of the P6 in the males, where the inner spine is particularly short in comparison with the outer seta, a characteristic that separates this species from other congeners such as *Eucyclops delachauxi*, *Eucyclops prionophorus* and *Eucyclops pseudoensifer*. The specimens depicted in Collado et al. (1984) show an inner spine which is at least twice as long as the outer seta, thus diverging from *Eucyclops bondi*. The comparison of structures present in the males has been useful to separate species of other Eucyclopinae, especially in *Paracyclops* (Karaytug 1999, Karaytug and Boxshall 1999). So, the inclusion of male characters, such as the structure and armature of P6 and the presence of aesthetascs and modified setae on the male antennules appears to be a valuable tool in the separation of the species of *Eucyclops*. Such characters should be incorporated in the current taxonomy of the genus. In Mexico there are more than 70 records of *Eucyclops bondi* (Suárez-Morales et al. 1996; Grimaldo-Ortega et al. 1998; Suárez-Morales and Reid 1998; Elías-Gutiérrez et al. 2008; Mercado-Salas 2009; Suárez-Morales and Walsh 2009; Mercado-Salas and Suárez-Morales 2012; Gutiérrez-Aguirre and Cervantes-Martínez 2013). In order to clarify the taxonomic status of these specimens we reviewed most of the Mexican records and we can only confirm the presence of a similar form of the male of *Eucyclops bondi* in a single locality in the central state of Aguascalientes that we will discuss in a forthcoming paper about the Mexican fauna of *Eucyclops*. The remaining records should be revised and it is probable that many will have to be reassigned to different species. *Eucyclops tziscao*, a species from southeast Mexico that is closely related to *Eucyclops bondi*, was described recently; this could represent one of the species to which some of the Mexican records of *Eucyclops bondi* could be assigned (Gutiérrez-Aguirre et al. 2013). Other records of *Eucyclops bondi* from the Americas that included drawings, but only of the females, are by Reid (1992), Suárez-Morales et al. (1996), Grimaldo-Ortega et al. (1998), and Elías-Gutiérrez et al. (2008). We looked for the main characteristics of the species in the drawings and concluded that the only record safely assignable to *Eucyclops bondi* is the one by Reid (1992) from Florida, but we consider that the male should be reviewed in order to confirm the species. In our opinion, the other records (Suárez-Morales et al. 1996; Grimaldo-Ortega et al. 1998; Elías-Gutiérrez et al. 2008) do not belong to *Eucyclops bondi* because some important differences were found. For instance, the dorsal seta in *Eucyclops bondi* is always longer than the outermost caudal seta, while in all other records from Mexico this seta presents an opposite condition, a dorsal seta shorter than the outermost caudal seta. We also detected additional differences, such as the presence of modified setae on the endopod and exopod of the fourth swimming leg in Grimaldo-Ortega et al. (1998) and the presence of hair-like spinules on the distal margin of the intercoxal sclerite, while the true *Eucyclops bondi* has spines on its distal margin.

### *Eucyclops leptacanthus* Kiefer, 1956

Figs 18–21

*Eucyclops leptacanthus*, Kiefer 1956

*Eucyclops* cf. *leptacanthus*, Reid 1993

*Eucyclops leptacanthus*, Mercado-Salas 2009

**Kiefer’s description.**

Total length of the single female found 0.76 mm, excluding caudal setae. The caudal ramus exactly four times as long as wide (82.5µ: 20.5µ). Position and shape of the two rami can be seen in Fig. 25. Inner margin is naked, outer margin provided with a “*serra*”, extending along almost the entire length of ramus, proximal spinules very small, very long spinules at the height of insertion of lateral seta, practically setiform. Measurements of setae of caudal rami from the innermost to outermost: 80µ, 356µ, 198µ, 60µ, length of dorsal seta about 45–50 µ; the ratio, based on the outermost terminal seta, is therefore 1.33: 5.93: 3.30: 1 (0.8). The two longest setae are quite weak and heteronomously plumose (Fig. 25).

Twelve-segmented antennules, margin of last three segments with a narrow hyaline membrane, margin of membrane finely denticulated (Fig. 26). Segmentation and setation of the swimming legs as usual, the final segment of the Enp of fourth leg with a length/width: 44.5µ: 20.5µ = 2.17. Inner apical spine about 55µ-56µ, notably longer than the length of segment and significantly longer than the outer spine which measures 35µ (Fig. 28). Segment of rudimentary leg elongated and bearing small and slender inner spine (Fig. 29). The seminal receptacle was not perceptible.

The male has not yet been found.

**Systematic position.** in the complex genus *Eucyclops*, where many forms exist that are similar to the single female specimen found here, a new species can only be established when the new form is distinguished by unique characteristics. The specimen of *Eucyclops* herein described possesses a well-developed *serra* on the caudal rami, a rudimentary leg with a slender inner spine, long apical spines on the last segment of endopod of fourth leg, as well as a finely denticulate hyaline membrane on the three last segments of the antennule, a combination of characters that I have not seen in any of the known *Eucyclops* species. For this reason I considered it as a new species and named as *Eucyclops leptacanthus* because of its slender spines on the fifth and fourth legs.

In the sample 10e I did find another *Eucyclops*-female. The hyaline membrane on the last segments of the antennules is equally finely denticulate; the *serra* on the caudal rami is not particularly remarkable, the inner spine of rudimentary leg is thicker, and the apical spine of the last segment of the endopodite of the fourth leg is wider than that in the above described species. This specimen must remain undetermined because of the few characters that could be seen. The same holds true in the two *Eucyclops* males from sample 11.

**Description based on Kiefer’s material.**

**Material examined.** Holotype. Adult ♀collected 03.11.1952 from Lake Orinoco, Barrancas, Venezuela (slides reference numbers SMNK05409, SMNK05410). Staatliches Museum für Naturkunde Karlsruhe, Germany.

**Figure 18.**
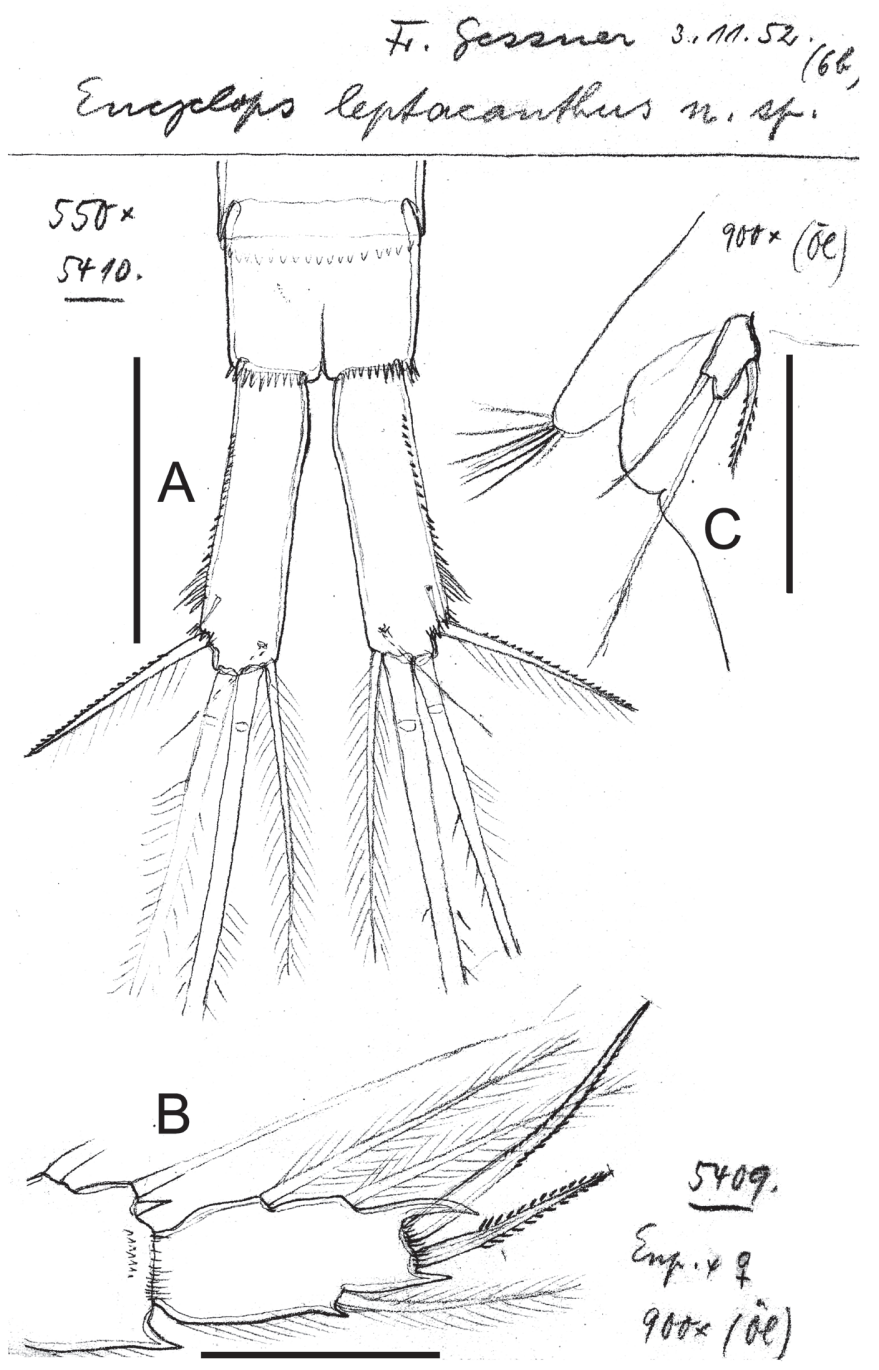
*Eucyclops leptacanthus* Kiefer, 1956. Original drawings of F. Kiefer. Female Holotype (two preparations same specimen) from Lake Orinoco, Venezuela. **A** Caudal rami **B** Enp3P4 **C** P5. Scale bars: **A** = 100 µm; **B–C** = 50 µm.

*Female*: Body length of holotype, excluding caudal setae, 760 µm. Urosome 5-segmented (Fig. 20A): relatively elongate; urosomal fringes smooth or weakly serrated. Genital double-somite symmetrical. Seminal receptacle typical of *serrulatus*-group, with rounded, lateral arms in posterior margin. Genital double somite about 1.3 times longer than wide. Length/width ratio of caudal ramus = 3.9–4.0; inner margin of caudal ramus naked; outer margin with strong spinules covering 63–64% of segment length, spinules distally increase in size (Figs 18A, 20A). Dorsal seta (VII) short, 0.5 times of length of caudal ramus, and 0.7 times as long as outermost caudal seta (III). Ratio of innermost caudal seta (VI)/outermost caudal seta (III) = 1.3–1.4. Lateral caudal seta (II) inserted at 74% of caudal rami. All terminal caudal setae plumose.

**Figure 19.**
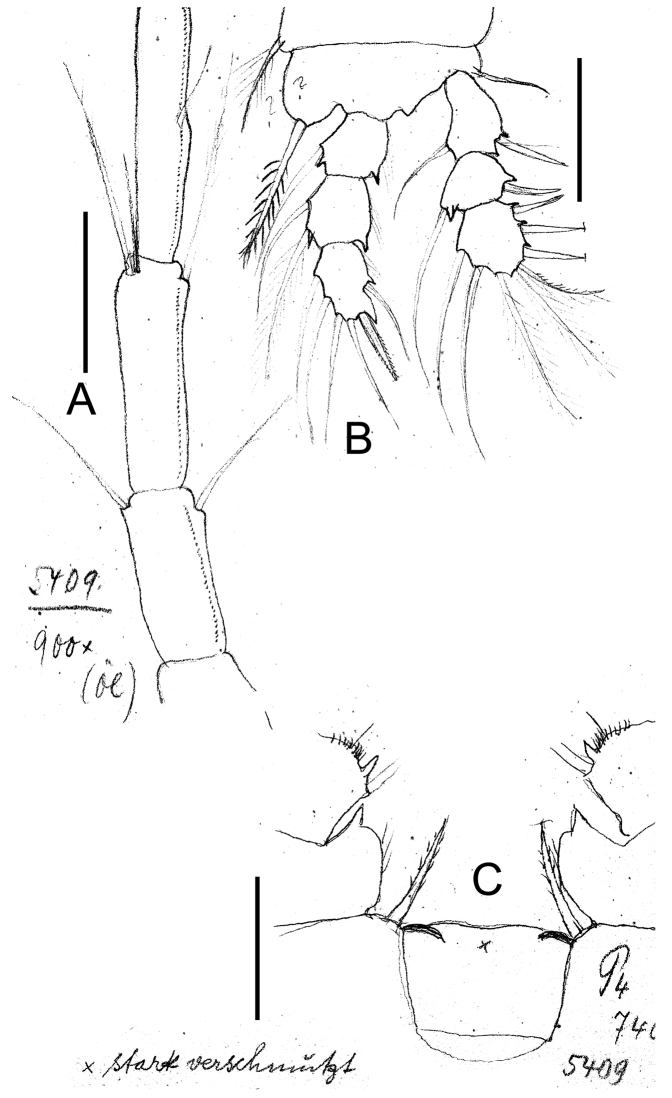
*Eucyclops leptacanthus* Kiefer, 1956. Original drawings of F. Kiefer. Holotype (two preparations same specimen) from Lake Orinoco, Venezuela. **A** Last segments of antennule **B** P1 **C** Intercoxal sclerite P4. Scale bars: **A–C** = 50 µm.

**Figure 20.**
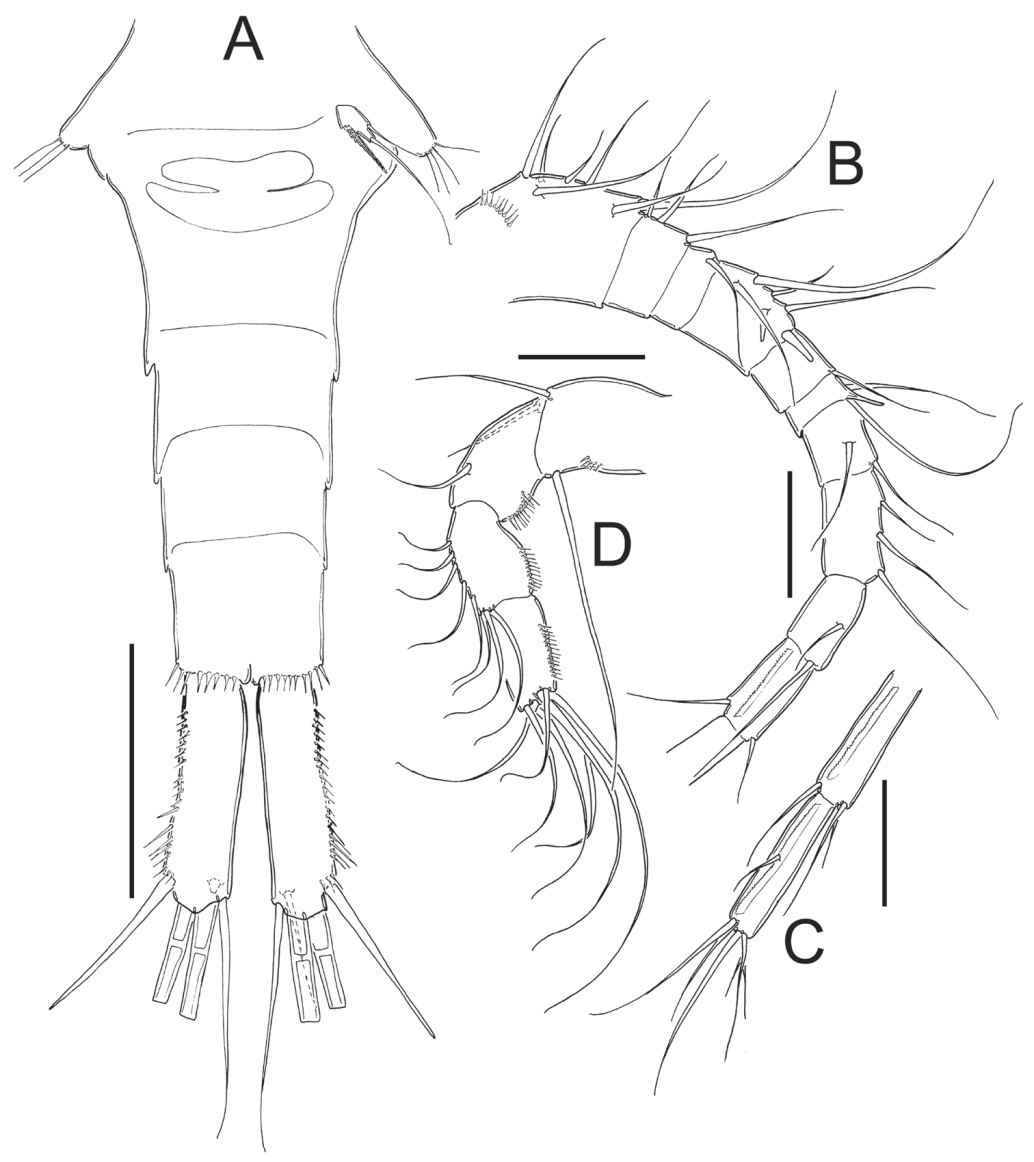
*Eucyclops leptacanthus* Kiefer, 1956. Authors’ drawings. Female Holotype from Lake Orinoco, Venezuela. **A** Urosome, ventral view **B** Antennule, segments 1–10 **C** Antennule, segments 11–12 **D** Antenna. Scale bars: **A** = 100 µm, **B–D** = 50 µm.

*Antennule* (Figs 19A, 20B–C): 12-segmented. Armament per segment as follows (s = seta, ae = aesthetasc, sp = spine): **1**(7s), **2**(3s), **3**(1s), 4(6s), **5**(1s), 6 (1s+1sp), 7(2s), 8(3s), **9**(2s), 10(2s), 11(3s), **12**(7s). Numbers in boldface indicate segments with incomplete ornamentation.

*Antenna* (Fig. 20D): Basis (2s + Exp), 3-segmented End (1s, 8s and 7s). Only row N17 was observable on basis of the holotype.

**Figure 21.**
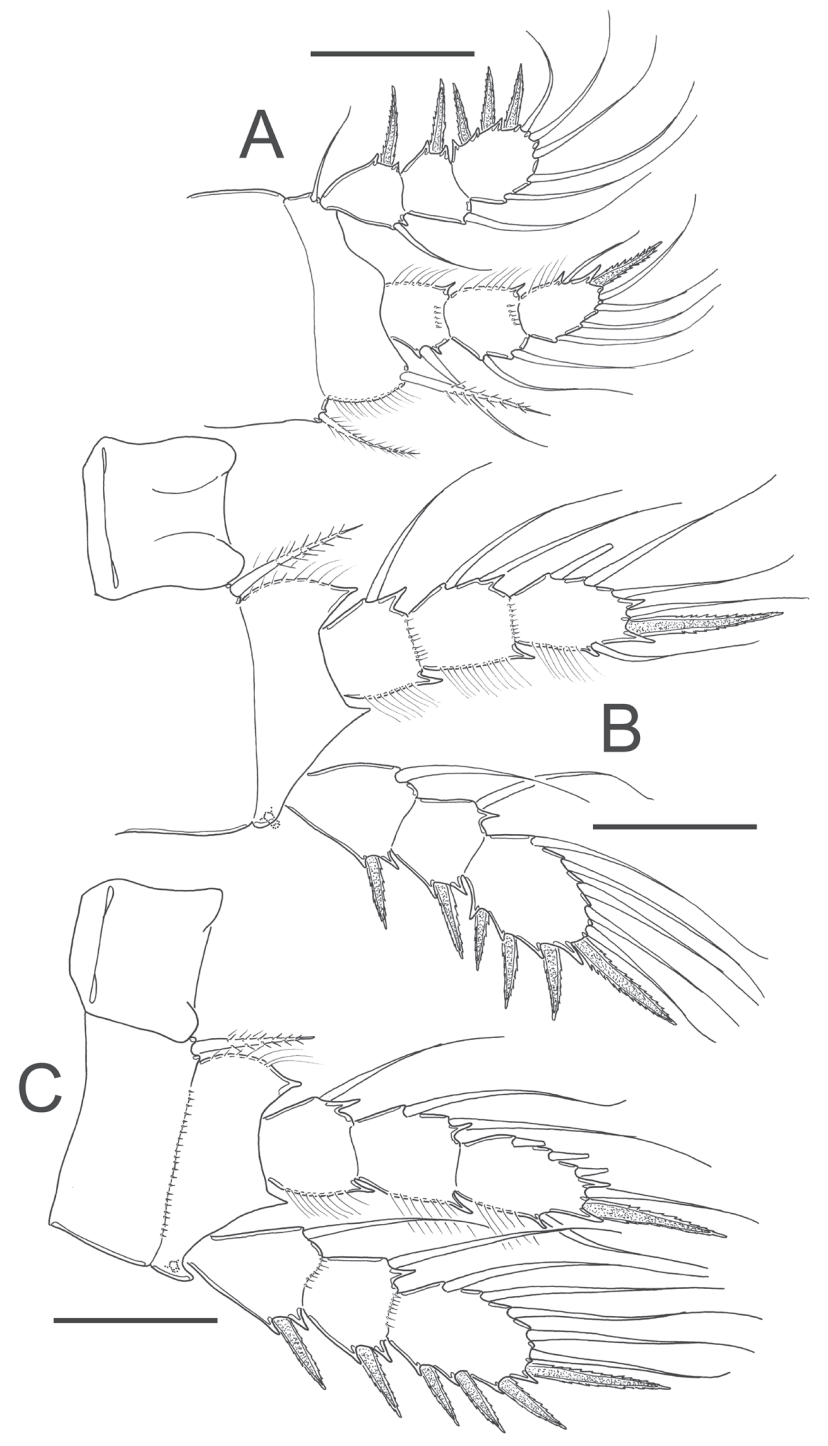
*Eucyclops leptacanthus* Kiefer, 1956. Authors’ drawings. Female Holotype from Lake Orinoco, Venezuela. **A** P1 **B** P2 **C** P3. Scale bars: **A–C** = 50 µm.

*Mouthparts*: not observable in the slide.

*P1–P4*: Endopods and exopods of all swimming legs three-segmented. Armature formula of all swimming legs as in Table 1.

*Leg 1* (Figs 19B, 21A): Coxa with strong, biserially setulated inner coxal seta. Basipodal spine not reaching middle of Enp3; and 0.7 times as long as endopodal ramus. Enp3 1.4–1.7 times as long as wide, apical spine of Enp3 as long as segment Enp3 (1:1), apicalmost seta of Enp3 1.5–1.8 times longer than apical spine.

*Leg 2* (Fig. 21B): No ornamentation observed on intercoxal sclerite, distal margin with 2 rounded projections. Coxa with strong, biserially setulated inner coxal seta. Enp3 1.6 times longer than wide, apical spine on Enp3 1.3 times as long as segment, Exp3 2.2 times as long as wide, apical spines of Exp3 0.9 times as long as segment. No modified setae were observed.

*Leg 3* (Fig. 21C): No cuticular ornamentation was observed on intercoxal sclerite, distal margin with 2 rounded projections. Coxa with strong, biserially setulated inner coxal seta. Enp3 2.0 times longer than wide, apical spine on Enp3 1.1 times as long as segment, apical seta of Enp3 1.2 times as long as apical spine. Exp3 1.9 times as long as wide, apical spine of Exp3 as long as segment (1:1). No modified setae observed.

*Leg*. *4* (Figs 18B, 19C): Intercoxal sclerite could not be clearly observed. Inner coxal spine with heteronomous setulation: basally with long hairs yet distally with spinules; lateral edge of inner coxal spine with 2 apical spine-like setules, proximal surface naked. Length/width ratio Enp3P4 = 2.3; length ratio inner/outer spines of Enp3P4 =1.5; length ratio inner spine of Enp3P4/Enp3P4 = 1.3; length ratio outer spine of Enp3P4/Enp3P4 = 0.8-0.9. Lateral seta of Enp3P4 inserted at 67-70% of total length of segment. No modified setae were observed.

*Leg 5* (Figs 18C, 20A): Free segment subrectangular, 1.8 times as long as wide bearing 1 inner spine and 2 setae; median seta longer than outer seta (about 1.8 times) and inner spine (about 2.6 times). Inner spine noticeably slender, 1.3 times as long as segment.

**Remarks.** *Eucyclops leptacanthus* is another species described by Kiefer (1956) that has been recorded from Mexico. Additional American records are from Costa Rica, Venezuela and Brazil (Rocha and Botelho 1998; Gutiérrez-Aguirre and Suárez-Morales 2001; Suárez-Morales 2004; Elías-Gutiérrez et al. 2008; Mercado-Salas 2009). This species is distinguished from the American congeners by the possession of a very slender spine on the female fifth leg and unmodified setae on P1–P4, the setae and spines of P1-P4 are relatively longer and narrower than in other species. Another characteristic of *Eucyclops leptacanthus* is the remarkably long innermost caudal seta, it is 1.3–1.4 times longer than outermost caudal seta and as long or slightly shorter than length of caudal ramus. In addition, the basipodal seta is distinctive in this species; when compared to some related species, it is short, reaching the proximal margin of P1 Enp3 whereas this seta reaches at least the middle of this segment in most American species of *Eucyclops*. A character that we couldn’t examine in the type material was the ornamentation of the P4 intercoxal sclerite because, as Kiefer stated on his drawings (Fig. 19C), the structure was very dirty. Nonetheless, we did note that differing from other species the plate was expanded horizontally, clearly wider than long. From the drawings presented by Collado et al. (1984), the Costa Rican specimens seem to agree with Kiefer’s description, but some differences have been detected. The fifth leg presents an outer seta that is only slightly shorter than the median seta, while in Kiefer’s description the outer seta is clearly shorter than the median seta. Also, the individuals from Costa Rica have short setae on Enp3 of P4, whereas these setae are long and slender in the type material (as mentioned above). The rest of the records from America did not include drawings that would allow further comparisons.

## Discussion

Among the 108 species and subspecies currently known in the genus *Eucyclops*, 28 are distributed in the Americas, most of the records in the continent are from surveys in the Eastern United States, Mexico, Argentina and Brazil (Reid 1985; Suárez-Morales 2004; Bruno et al. 2005; Frisch and Threlkeld 2005; Alekseev et al. 2006; Gaviria and Aranguren 2007; Elías-Gutiérrez et al. 2008; Suárez-Morales and Walsh 2009; Mercado-Salas 2009; Suárez-Morales et al. 2010; De los Ríos et al. 2010; Mercado-Salas et al. 2012). Due to its diversification in different geographic regions and the taxonomical problems within the genus, it is likely that the fauna of *Eucyclops* in the Americas has been underestimated. Our analysis of the records of four of the 28 species recorded in the Americas revealed that many published records of these species are actually not assignable to these species and should be compared and re-checked using upgraded descriptive standards in order to clarify their taxonomic and biogeographic status. In addition, if we consider that about 40% of the records in the continent have been assigned to taxonomically complex and widely distributed taxa such as *Eucyclops serrulatus*, *Eucyclops agilis*, and *Eucyclops speratus*, we can have a general idea about how the diversity of the genus is underestimated in the Americas.
